# The blood proteome of imminent lung cancer diagnosis

**DOI:** 10.1038/s41467-023-37979-8

**Published:** 2023-06-01

**Authors:** The Lung Cancer Cohort Consortium (LC3), Demetrius Albanes, Karine Alcala, Nicolas Alcala, Christopher I. Amos, Alan A. Arslan, Julie K. Bassett, Paul Brennan, Qiuyin Cai, Chu Chen, Xiaoshuang Feng, Neal D. Freedman, Florence Guida, Rayjean J. Hung, Kristian Hveem, Mikael Johansson, Mattias Johansson, Woon-Puay Koh, Arnulf Langhammer, Roger L. Milne, David Muller, Justina Onwuka, Elin Pettersen Sørgjerd, Hilary A. Robbins, Howard D. Sesso, Gianluca Severi, Xiao-Ou Shu, Sabina Sieri, Karl Smith-Byrne, Victoria Stevens, Lesley Tinker, Anne Tjønneland, Kala Visvanathan, Ying Wang, Renwei Wang, Stephanie Weinstein, Jian-Min Yuan, Hana Zahed, Xuehong Zhang, Wei Zheng

**Affiliations:** 1 Metabolic Epidemiology Branch, Division of Cancer Epidemiology and Genetics, National Cancer Institute, Rockville, MD USA; 2 Genomic Epidemiology Branch, International Agency for Research on Cancer, Lyon, France; 3 Institute for Clinical and Translational Research, Baylor College of Medicine, Houston, TX USA; 4 Department of Population Health, New York University School of Medicine, New York, NY USA; 5 Cancer Epidemiology Division, Cancer Council Victoria, Melbourne, VIC Australia; 6 Vanderbilt University School of Medicine, Nashville, TN USA; 7 Public Health Sciences Division, Fred Hutchinson Cancer Research Center, Seattle, WA USA; 8 Division of Cancer Epidemiology and Genetics, National Cancer Institute, Rockville, MD USA; 9 Environment and Lifestyle Epidemiology Branch, International Agency for Research on Cancer, Lyon, France; 10 Prosserman Centre for Population Health Research, Lunenfeld-Tanenbaum Research Institute, Sinai Health, Toronto, ON Canada; 11 Dalla Lana School of Public Health, University of Toronto, Toronto, ON Canada; 12 HUNT Research Centre, Norwegian University of Science and Technology, Levanger, Norway; 13 Department of Radiation Sciences, Oncology, Umea University, Umea, Sweden; 14 Healthy Longevity Translational Research Program, Yong Loo Lin School of Medicine, National University of Singapore, Singapore, Singapore; 15 Singapore Institute for Clinical Sciences, Agency for Science Technology and Research (A*STAR), Singapore, Singapore; 16 Department of Public Health and Nursing, Norwegian University of Science and Technology, Levanger, Norway; 17 Centre for Epidemiology and Biostatistics, The University of Melbourne, Melbourne, VIC Australia; 18 Precision Medicine, School of Clinical Sciences at Monash Health, Monash University, Clayton, NC Australia; 19 Division of Genetic Medicine, Imperial College London School of Public Health, London, UK; 20 Brigham and Women’s Hospital, Harvard Medical School, Boston, MA USA; 21 Inserm, Université Paris-Saclay, Villejuif, France; 22 Vanderbilt University Medical Center, Nashville, TN USA; 23 Epidemiology and Prevention Unit, Fondazione IRCCS Istituto Nazionale dei Tumori, Milan, Italy; 24 Cancer Epidemiology Unit, University of Oxford, Oxford, UK; 25 Rollins School of Public Health, Emory University, Atlanta, GA USA; 26 Women’s Health Initiative Clinical Coordinating Center, Fred Hutchinson Cancer Research Center, Seattle, WA USA; 27 Diet, Cancer and Health, Danish Cancer Society Research Center, Copenhagen, Denmark; 28 Department of Epidemiology, Johns Hopkins Bloomberg School of Public Health, Baltimore, MD USA; 29 American Cancer Society, Atlanta, GA USA; 30 UPMC Hillman Cancer Center, University of Pittsburgh, Pittsburgh, PA USA; 31 Department of Epidemiology, University of Pittsburgh, Pittsburgh, PA USA

**Keywords:** Predictive markers, Non-small-cell lung cancer, Tumour biomarkers

## Abstract

Identification of risk biomarkers may enhance early detection of smoking-related lung cancer. We measured between 392 and 1,162 proteins in blood samples drawn at most three years before diagnosis in 731 smoking-matched case-control sets nested within six prospective cohorts from the US, Europe, Singapore, and Australia. We identify 36 proteins with independently reproducible associations with risk of imminent lung cancer diagnosis (all *p* < 4 × 10^−5^). These include a few markers (e.g. CA-125/MUC-16 and CEACAM5/CEA) that have previously been reported in studies using pre-diagnostic blood samples for lung cancer. The 36 proteins include several growth factors (e.g. HGF, IGFBP-1, IGFP-2), tumor necrosis factor-receptors (e.g. TNFRSF6B, TNFRSF13B), and chemokines and cytokines (e.g. CXL17, GDF-15, SCF). The odds ratio per standard deviation range from 1.31 for IGFBP-1 (95% CI: 1.17–1.47) to 2.43 for CEACAM5 (95% CI: 2.04–2.89). We map the 36 proteins to the hallmarks of cancer and find that activation of invasion and metastasis, proliferative signaling, tumor-promoting inflammation, and angiogenesis are most frequently implicated.

## Abstract (web summary)

Lung cancer screening could enhance early diagnosis and treatment. Here, the authors used proteomic analysis of pre-diagnosis samples across 6 cohorts to identify 36 proteins associated with imminent lung cancer diagnosis.

## Introduction

Lung cancer is the leading cause of cancer death globally^1^. The 5-year survival is 20%, but varies from 60% for early-stage disease (Stage 1-2) to 6% for late-stage disease (stage 4)^2^. In the United States (US), lung cancer mortality declined by 6% annually from 2013 to 2016^3^. This improvement can be attributed to advancements in diagnosis and treatment for patients with both early- and late-stage lung cancer^4^. Improved surgical techniques, including stereotactic body radiotherapy (SBRT) and adjuvant chemotherapy, have improved prognosis for early-stage patients, whereas patients with locally advanced disease have benefitted from the introduction of radio-chemotherapy, adjuvant immunotherapy, and neoadjuvant immune checkpoint inhibitors (ICIs). However, most lung cancer patients are diagnosed with late-stage disease where curative treatment is rarely possible, even though developments in targeted and immunotherapy combinations have improved short-term survival^4^.

Despite advances in lung cancer treatment, improving early detection is the most promising strategy to improve long-term survival. Screening with low-dose computed tomography (LDCT) has the potential to substantially increase the proportion of lung cancer patients diagnosed with early-stage disease who can be offered treatment with curative intent. The ability of LDCT screening to decrease lung cancer mortality among high-risk people with a history of smoking has been demonstrated in several randomized trials^5,6^, but some concerns remain, including how to best identify and reach those individuals who are likely to benefit from screening, and how to manage indeterminate pulmonary nodules detected on LDCT.

The advent of LDCT screening and the introduction of targeted therapies have highlighted a need to identify lung cancer biomarkers that can be used to (i) identify high-risk individuals who may benefit from screening, (ii) inform diagnostic work-up and nodule management after LDCT screening, and (iii) choose optimal treatment regimens and monitor response to treatment. In 2018, the US National Cancer Institute funded the Integrative Analysis of Lung Cancer Etiology and Risk (INTEGRAL) program, an ambitious initiative focusing on developing biomarkers that can refine eligibility criteria for LDCT screening and diagnostic work-up following LDCT^7^. Here, we present results from the initial large-scale analysis designed to identify circulating protein biomarkers associated with imminent lung cancer diagnosis in the general population of individuals with a smoking history. Using a high-throughput proteomics approach, we screened over 1000 circulating proteins in blood samples drawn up to three years prior to diagnosis within the Lung Cancer Cohort Consortium (LC3).

We here focus on identifying proteins robustly associated with risk of imminent lung cancer diagnosis, and then describing their epidemiological properties, the biological pathways to which they belong, and their known relevance in carcinogenesis.

## Results

Our study was designed to identify protein markers of imminent lung cancer in people with a smoking history from the general population. We defined imminent lung cancer as a clinical lung cancer diagnosis within three years of blood draw and identified 731 lung cancer cases and 731 smoking-matched controls in six prospective cohort studies from the LC3 consortium.

Most study participants were men (980 men vs. 482 women) and the mean age at blood collection was 65 years (standard deviation 9 years). The mean time between pre-diagnostic blood collection and diagnosis was 1.6 years (range: 0–3 years, by design) (Table 1). Demographic characteristics stratified by cohort are presented in Supplementary Data 1.

**Table 1:** Characteristics of 731 lung cancer cases and 731 matched controls from the Lung Cancer Cohort Consortium included in analyses to identify protein biomarkers of imminent lung cancer diagnosis

Characteristic	Cases	Controls
	*N* (%) or mean (SD)	*N* (%) or mean (SD)
Total number of participants	731	731
Female	241 (33%)	241 (33%)
Age, years	64.8 (9.1)	64.7 (9.2)
Body mass index, kg/m^2^	25.5 (4.2)	26.2 (4.3)
Follow-up time, years^a^	1.6 (0.9)	11.9 (5.4)
Follow-up survival time, years^b^	4.1 (4.1)	13.0 (5.4)
Smoking status		
Current	397 (54%)	400 (55%)
Former	334 (46%)	331 (45%)
Cigarettes smoked per day	20.9 (13.3)	16.3 (11.7)
Years smoked	39.5 (12.2)	36.2 (14.0)
Years since cessation, among former	15.4 (11.7)	19.0 (13.6)
Histology		
Adenocarcinoma	246 (34%)	
Squamous cell carcinoma	150 (20%)	
Large cell carcinoma	27 (4%)	
Small cell carcinoma	118 (16%)	
Other/NOS	190 (26%)	
Stage		
Early stage (TNM 1/2)	78 (23%)	
Late stage (TNM 3/4)	256 (77%)	
Unknown/missing	397	
Participating cohort		
CPS	115 (16%)	115 (16%)
EPIC	188 (26%)	188 (26%)
HUNT	164 (22%)	164 (22%)
MCCS	108 (15%)	108 (15%)
NSHDS	64 (9%)	64 (9%)
SCHS	92 (12%)	92 (12%)

^a^Time from blood draw to end of follow-up or lung cancer diagnosis.

^b^Time between blood draw and the end of follow-up for mortality (including death).

Age, body mass index, and smoking information is assessed at the time of blood draw.

*EPIC* The European Prospective Investigation into Cancer and Nutrition, *NSHDS* Northern Sweden Health and Disease Study, *HUNT* The Trøndelag Health Study, *MCCS* The Melbourne Collaborative Cohort Study, *SCHS* The Singapore Chinese Health Study, *CPS-II* The Cancer Prevention Study II.

### Identification and description of proteins associated with imminent lung cancer

We used the Olink Proteomics (https://www.olink.com/) platform to measure relative concentrations of up to 1162 individual proteins across 14 panels. We initially measured all available panels in samples from 252 case-control pairs selected from the European Prospective Investigation into Cancer and Nutrition (EPIC) study and the Northern Sweden Health and Disease Study (NSHDS). Subsequently, among 479 additional case-control pairs selected from four additional cohorts, we re-measured a subset of protein panels (totalling between 392 and 484 proteins), which were chosen to maximize coverage of the proteins with the strongest risk associations (Supplementary Table 1). Controls were matched to cases by age, date of blood draw, sex, cohort, and smoking information in four categories (details in Methods section). Quality control results are provided in Supplementary Data 2a, b and 3. For statistical analyses, we replaced protein measurements below the lower limit of detection (LOD) with LOD/$\surd 2$ according to the manufacturer’s recommendation.

### Overall discovery analysis of proteins associated with lung cancer risk

**Fig. 1:**
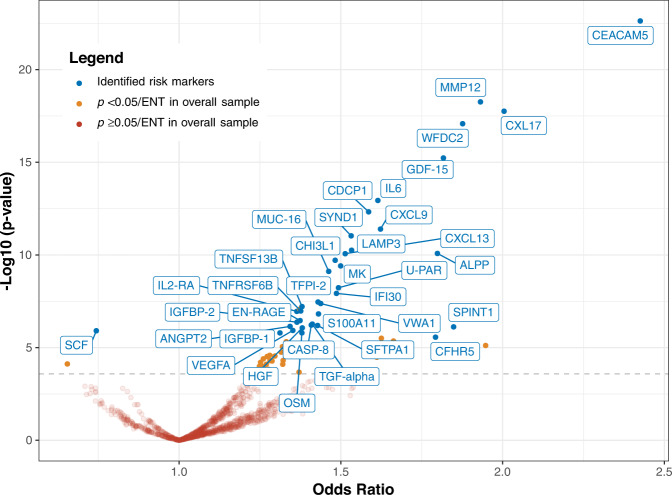
Identification of 36 protein biomarkers associated with risk of imminent lung cancer diagnosis among 731 cases and 731 matched controls in the Lung Cancer Cohort Consortium. The volcano plot depicts the lung cancer odds ratio per standard deviation increment in relative protein concentrations (log-base-2 transformed) (*x* axis) and the −Log10 *p* value (*y* axis). The 36 identified markers of imminent lung cancer are labeled (see Methods; markers were identified through a resampling process that measured the association of each protein with lung cancer risk in a discovery set and a replication set. The risk markers were required to have a *p* < 0.05/effective-number-of-tests in the discovery set and *p* < 0.05 in the replication set in at least 50% of the resampling iterations). Source data are provided as a Source Data file.

We evaluated the association of each protein with risk of imminent lung cancer diagnosis using conditional logistic regression models. The associations between all 1162 proteins and lung cancer risk are reported in Fig. 1 and Supplementary Data 4. In the full study sample, there were 67 proteins associated with lung cancer after accounting for multiple comparisons using the effective-number-of-tests method^8^ (Supplementary Data 4). We subsequently implemented a resampling procedure to simulate 500 iterations of an independent discovery-replication design, which was designed to more stringently identify proteins whose associations with lung cancer had high reproducibility. As intended, the resampling algorithm identified a smaller group of 36 proteins (Fig. 1, Supplementary Figs. 1 and 2, Supplementary Data 5). A flow chart depicting this analysis is presented in Supplementary Fig. 1.

Among the 36 markers identified by the resampling algorithm, all but one (SCF) were positively associated with lung cancer risk (Fig. 1). Among these, the estimated odds ratio per standard deviation (OR_sd_) ranged from 1.31 (IGFBP-1, 95% confidence interval [95% CI]: 1.17–1.47, *p* = 2 × 10^−6^) to 2.43 (CEACAM5, 95% CI: 2.04–2.89, *p* = 2 × 10^−23^) (Supplementary Data 4). The SCF protein was negatively associated with lung cancer (OR = 0.74, 95% CI: 0.66–0.84, *p* = 1.24 × 10^−6^). Compared with the PLCOm2012 model^9^, a well-performing prediction model for smoking-related lung cancer which uses questionnaire information, the individual proteins improved discrimination between future lung cancer cases and controls by between 0.005 (OSM) and 0.082 (CEACAM5) units in the area under the receiver operating curve (AUC) (Supplementary Data 4). All 36 proteins showed good quality control measures and had less than 20% of values below LOD (Supplementary Data 2, Supplementary Data 3).

In a sensitivity analysis, we compared the proteins that would be identified if we used a single split-sample approach for discovery and replication instead of our resampling algorithm (details in Methods section). This showed that there were 29 proteins identified by both methods, 7 markers identified only by the resampling algorithm, and 10 markers identified only by the single split-sample method (Supplementary Fig. 3). Markers identified only by the resampling algorithm typically had stronger risk associations in the full dataset and were more consistently associated with risk across the six cohorts compared with the proteins identified only by the single split-sample method (Supplementary Data 6).

For the 36 proteins identified by the resampling algorithm as having replicable associations with risk of imminent lung cancer diagnosis, the following results describe their epidemiological and gene expression characteristics, as well as their known relevance in carcinogenesis.

### Analyses considering stage at diagnosis, histological subtype, and lead time

Among cases with complete stage information at diagnosis, 256 of 334 cases were diagnosed at late stage (stage 3–4) (Table 1). A majority of proteins (23 out of 36) showed stronger odds ratios for late-stage compared with early-stage (stage 1–2) lung cancer, but a clear difference (*p*-heterogeneity [*p*_het_] < 0.05) was only apparent for two proteins (CXL17 and CEACAM5) (Supplementary Data 7, Supplementary Fig. 4). Stage-stratified odds ratio and AUC estimates are presented in Supplementary Data 7. For the subset of lung cancer cases with available information on stage at diagnosis, we estimated the stage at blood draw using sojourn times specific to stage, histological type, and sex previously estimated by ten Haaf et al.^10^. This suggested that 78% of cases were likely early stage (stage 2 or earlier) at the time of blood draw (Supplementary Fig. 5).

In Supplementary Data 8, we present associations between the 36 identified proteins and lung cancer risk by the major histological subtypes and demographic strata (sex, smoking status, cohort, and lead time). Most of the markers displayed consistent risk associations across the major histological subtypes. Exceptions (*p*_het_ < 0.05) included CEACAM5, which was more strongly associated with adenocarcinoma than squamous cell carcinoma, and MMP12, which was more strongly associated with squamous cell carcinoma than with adenocarcinoma (Supplementary Data 8, Supplementary Fig. 6).

When stratifying by lead time (time between blood draw and diagnosis), 19 proteins showed heterogeneity in associations (*p*_het_ < 0.05, Supplementary Data 8) and 11 had a clear trend in the strength of association across categories of lead time (*p*_trend_ < 0.05, Supplementary Fig. 7, Supplementary Data 9). For instance, EN-RAGE displayed little evidence for an association with lung cancer at 2–3 years prior to diagnosis (OR_2–3y_: 1.10, 95% CI: 0.91–1.33), but was strongly associated within one year of diagnosis (OR_<1y_: 2.49, 95% CI: 1.87–3.32, *p*_het_ = 6 × 10^−6^). A similar pattern was observed for IL6 (OR_2–3y_: 1.36, 95% CI: 1.10–1.67 vs OR_<1y_: 2.56, 95% CI: 1.92–3.41, *p*_het_ < 0.001).

### Analyses considering smoking history and demographic factors

**Fig. 2:**
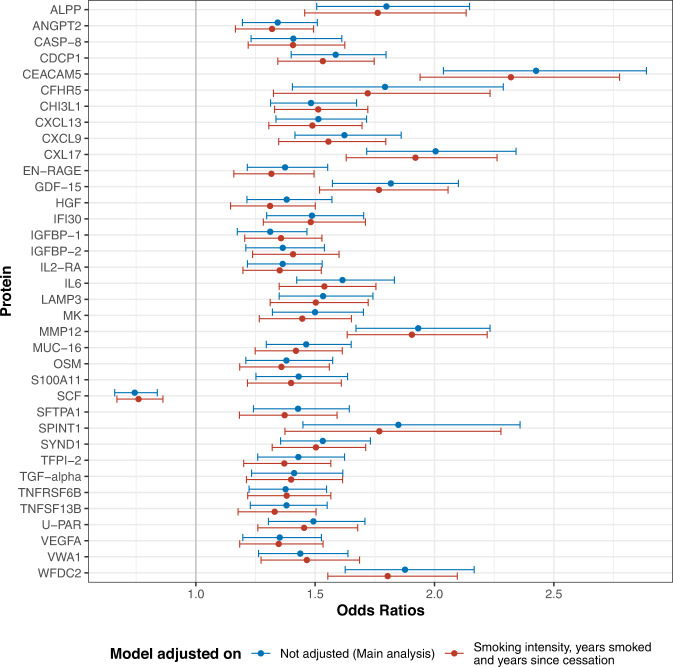
Lung cancer odds ratios for the 36 proteins associated with imminent lung cancer diagnosis before and after detailed adjustment for smoking intensity, duration, and years since cessation. Data for 95% confidence intervals are presented as$\,{e}^{(\beta \pm 1.96\times {sd})}$. *β* is the estimate from each conditional logistic regression, and *sd* is their respective standard deviation. Number of samples used are presented in Supplementary Data 10. Source data are provided as a Source Data file.

Stratified analysis by smoking status highlighted two proteins, IGFBP-1 and VWA1, that had stronger lung cancer risk associations in current vs former smokers (*p*_het_ < 0.05, Supplementary Data 8, Supplementary Fig. 8). Additionally, accounting for smoking intensity, duration and years since cessation resulted in very little attenuation of the OR estimates (Fig. 2, Supplementary Data 10). When evaluating cross-sectional relationships between protein concentrations and smoking history metrics in controls using linear regression adjusted for sex, age and cohort, we found that many markers had different concentrations when comparing former and current smokers, but only GDF-15 was associated with smoking intensity after accounting for multiple comparisons (Supplementary Fig. 9a). We also found SCF inversely associated with smoking duration. When analyzing lung cancer cases and controls combined (whilst additionally accounting for case-control status), we found several additional proteins associated with smoking intensity and duration (Supplementary Fig. 9b).

Further risk analyses stratified by demographic factors did not identify important heterogeneity in associations (Supplementary Data 8). However, in a separate exploratory analysis in the SCHS cohort, whose participants are of Han-Chinese descent, we found two proteins, RFNG and S100A4, associated with lung cancer risk (*p* < 0.05/effective-number-of-tests), despite showing little evidence for an association among participants of European, US, or Australian cohorts (Supplementary Fig. 10). The OR_sd_ for RFNG in SCHS was 2.65 (95% CI: 1.62–4.33, *n* case sets: 90) compared with 1.07 (95% CI: 0.93–1.23, *n* case sets: 455) in the other cohorts (*p*_het_ < 0.001), and the OR_sd_ for S100A4 in SCHS was 2.77 (95% CI: 1.72–4.44, *n* case sets: 92) compared with 1.03 (95% CI: 0.90–1.18, *n* case sets: 620) in the other cohorts (*p*_het_ < 0.001).

### Relationships between risk proteins and their role in cancer development

**Fig. 3:**
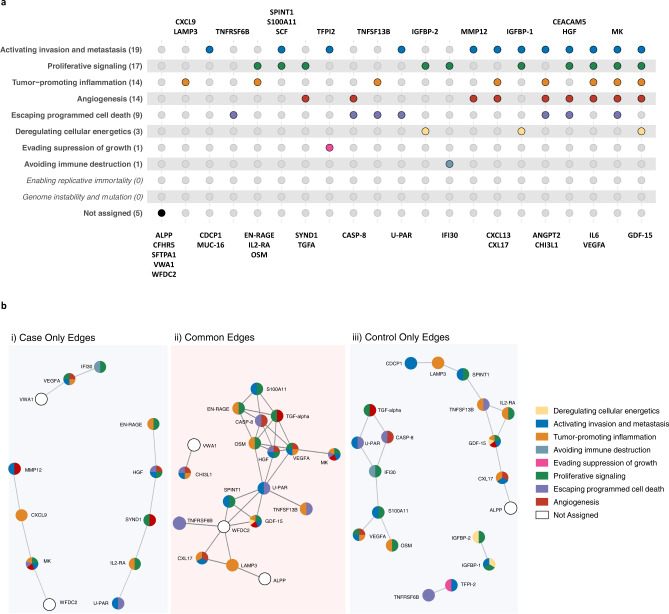
Biological context of the 36 proteins associated with risk of imminent lung cancer diagnosis. **a** Relationship between our 36 proteins and the 10 hallmarks of cancer described by Hanahan and Weinberg, based on their descriptions and functions available on GeneCards, the Human Protein Atlas, and Uniprot. Each hallmark is represented by a different color. **b** Network analysis among the 36 proteins, the figure depicts partial correlation networks (accounting for sex, age, cohort, and all other identified proteins) and stable protein associations. In lung cancer cases, no stable connections were found for ANGPT2, CDCP1, CEACAM5, CFHR5, CXCL13, IGFBP-1, IGFBP-2, IL6, MUC-16, SCF, SFTPA1, TFPI-2. In controls, no stable connections were found for ANGPT2, CEACAM5, CFHR5, CXCL13, CXCL9, IL6, MMP12, MUC-16, SCF, SFTPA1, and SYND1. Source data are provided as a Source Data file.

To contextualize the biological roles of the identified markers in cancer development, we assigned the proteins to one or more of the ten hallmarks of cancer as defined by Hanahan and Weinberg^11,12^ based on their description and functions available on GeneCards, the Human Protein Atlas, Uniprot^13–15^, and the pathways in which they are implicated according to g:profiler^16^. Among the 36 markers, we found that 31 had documented functions within the hallmarks of cancer (Fig. 3a). The most frequently implicated hallmark was “activating invasion and metastasis*”*, to which 19 proteins where assigned, including CEACAM5, MMP12, U-PAR and CDCP1. The second most frequently implicated hallmark was “proliferative signaling*”*, to which 17 proteins were assigned. We also found many proteins (*n* = 14) assigned to “angiogenesis*”* or “tumor promoting inflammation*”*. When using g:Profiler^16^ to query the list of genes that code for the identified proteins, we found that the most enriched pathways were “extracellular region”, “responses to stimulus” and “regulation of biological processes” (Supplementary Figs. 11 and 12, Supplementary Table 2).

To assess relationships between proteins, we first quantified pairwise correlations between the 36 identified risk proteins using adjusted Pearson correlation coefficients separately in cases and controls (Supplementary Fig. 13). Most proteins were moderately and positively correlated, except for SCF which was inversely correlated with some proteins (as well as with lung cancer risk, see above). These patterns were similar in cases and controls.

To consider the relationships among all proteins simultaneously, we implemented sparse graphical network models adjusted for partial correlations between proteins, separately in cases and controls (Fig. 3b). We found U-PAR to be the most highly connected and central protein in both the case and control networks (eight connections among cases and nine among controls, Supplementary Data 11). Although most protein connections were common to controls and cases, we found evidence for three distinct clusters of proteins with stable associations observed only among cases. One was centered around SYND1 [Cluster_1_: U-PAR, IL2-RA, SYND1, HGF, and EN-RAGE], one around VEGFA [Cluster_2_: VWA1, VEGFA and IFI30], and one around MK and CXCL9 [Cluster_3_: MMP12, CXCL9, MK, and WFDC2]. The Cluster_1_ network was enriched for markers related to inflammatory response (g:profiler pathway analyses *P*_adjusted_ = 7.4 × 10^−3^) and Cluster_3_ was enriched for proteins involved in homeobox six-3 transcription factor and defense and immune responses (g:Profiler *P*_adjusted_: 4 × 10^−2^, g:Profiler *P*_adjusted_: 3 × 10^−2^ and g:Profiler *P*_adjusted_: 4 × 10^−2^). Notably, several of the proteins most strongly associated with lung cancer, including CEACAM5, IL6, and SCF, were weakly correlated with other markers and did not have any stable connections with other identified risk markers (Fig. 3b).

### Associations with mortality among individuals with lung cancer

Using Cox proportional hazards models, we evaluated the extent to which the 36 risk proteins were associated with all-cause mortality following lung cancer diagnosis using both blood concentrations and tumor gene expression in TCGA samples. Whilst 20 proteins were nominally associated (*p* < 0.05) with all-cause mortality when measured in blood (Supplementary Fig. 14), these associations were weak in comparison to the association with incident lung cancer risk. Only three proteins (CEACAM5, CDCP1 and VEGFA) were associated with all-cause mortality after accounting for multiple comparisons (Supplementary Data 12 and 13). Of the 20 proteins nominally associated with mortality, three were also nominally associated with all-cause mortality when assessed using tumor gene expression (CDCP1, CEACAM5, and U-PAR) in TCGA.

### Gene expression in normal and tumor tissue

**Fig. 4:**
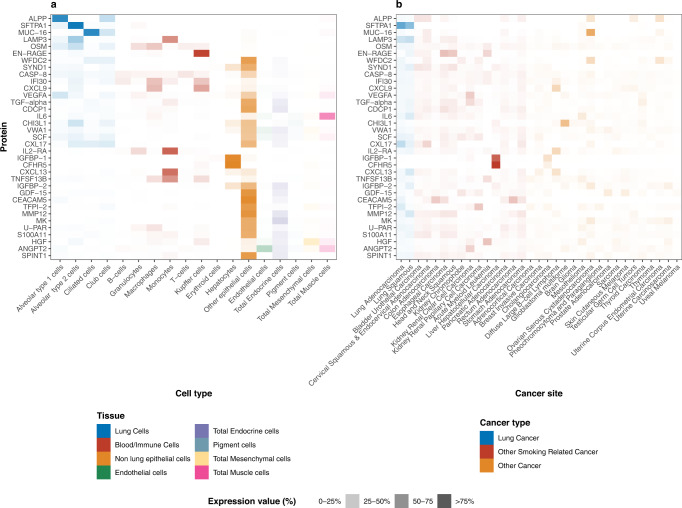
Gene expression of 36 protein biomarkers associated with risk of imminent lung cancer diagnosis in normal and tumor tissue. Proteins are listed in order of their relative expression in non-cancerous lung cell. **a** mRNA expression in normal tissue (gtex). **b** mRNA expression in tumor tissue (TCGA). Source data are provided as a Source Data file.

We used data from GTEx to assess mRNA expression for the genes coding for 36 risk proteins in normal tissue. Relative levels of mRNA expression in various normal cell types for 35 markers are shown in Fig. 4a (data was not available for TNFRSF6B). Three markers (ALPP, SFTPA1, and MUC-16) were expressed primarily by lung cell types, while 4 others (IL2-RA, CXCL13, TNFSF13B, and EN-RAGE) were expressed primarily in immune cells. For mRNA expression in tumor cell types from TCGA, we found that most of the 36 markers were expressed in lung tumor tissue to some degree, but also in a wide variety of other cancer types (Fig. 4b). The only marker that appeared specifically expressed in lung cancer tissue was SFTPA1.

## Discussion

The INTEGRAL project is a major initiative aiming to identify circulating protein biomarkers of imminent—but yet-to-be diagnosed—lung cancer. Based on blood samples drawn up to 3 years prior to clinical lung cancer diagnosis, we used a high-throughput proteomics platform to evaluate the association of up to 1162 circulating proteins with imminent lung cancer diagnosis in 731 cases and 731 matched controls from six prospective population cohorts. We identified 36 proteins associated with risk of imminent lung cancer diagnosis, most of which have not been previously identified as pre-diagnostic lung cancer biomarkers.

The last decade has seen major investments in research aiming to identify early cancer biomarkers. With the advent of early detection by LDCT screening, a strong focus has been placed on lung cancer. A wide array of circulating biomarkers have been proposed, including germline gene variants^17,18^, microRNA^19,20^, epigenetic markers^21^, autoantibodies^22^, protein markers^23,24^, and circulating tumor DNA^25^. However, few have been independently validated, and none are widely used in screening. In the INTEGRAL project, we decided to focus on circulating proteins due to their demonstrated ability to improve the discrimination of smoking-based risk prediction in an independent validation population^23,24^, as well as the prospect of developing a clinical biomarker test at a reasonable cost and sample volume requirement.

Our current study analyzed 1162 circulating proteins and found 67 proteins associated with lung cancer risk after accounting for multiple testing. Following a resampling algorithm to simulate many iterations of split-sample discovery and replication, we identified 36 proteins with replicable associations with risk of imminent lung cancer diagnosis, 35 of which showed positive associations with risk. Comparing results from the resampling algorithm vs. a single-split discovery/replication analysis demonstrated that our procedure for identifying proteins is conservative, thus allowing us to comfortably conclude that they are associated with risk of imminent lung cancer across the studied populations. Six of the 36 markers have been previously reported to be associated with lung cancer in pre-diagnostic samples, including several well-known tumor markers such as CEACAM5/CEA and CA-125/MUC-16^24^, as well as IL6, CDCP1, CXCL9 and CXCL13^26–28^.

We characterized the epidemiological properties of the identified proteins and their associations to known risk factors such as smoking. Despite several proteins being associated with smoking history cross-sectionally^29,30^, we found limited evidence for heterogeneity in risk associations for most of the 36 markers when stratifying by smoking status, and little impact of additional adjustment for smoking characteristics. However, we did find stronger risk associations for many of the 36 markers when measured in blood drawn closer to diagnosis. This is expected for markers indicative of forthcoming disease, as opposed to markers of disease etiology. Among these proteins, two markers from the S100 family (EN-RAGE and S100A11) displayed particularly strong associations closer to diagnosis. Proteins in the S100 family are implicated in tumorigenesis and cancer progression through different mechanisms of inflammation, cell differentiation, and cell proliferation^31^, and have been proposed as biomarkers for prognosis of melanoma^32,33^. These observations suggest that the risk associations are likely to reflect a somatic response to (or the direct action of) a subclinical lung tumor, rather than differences in tobacco exposure. Together with the risk discrimination analysis that indicated improvements over the PLCOm2012 model for several individual proteins, they also suggest that the identified markers provide additional risk information to that of detailed smoking history. We plan to evaluate the extent to which a combination of proteins may inform risk discrimination in a separate study. Of note, some markers did not display stronger risk associations closer to diagnosis, although we could only analyze trends over a maximum of 3 years lead time, by design. Future studies should therefore seek to describe patterns in risk associations for the identified markers over longer lead times.

A potential role for the identified protein markers in early detection of lung cancer is supported by our analysis estimating that 78% of cases with known stage at diagnosis were stage 2 or earlier at the time of blood draw, and 68% stage 1 or earlier, which suggests that the markers may be able to detect many lung cancers at a curable stage. Further, we observed improvements in risk discrimination when the proteins were individually added to the established PLCOm2012 smoking-based risk prediction model. We find these results encouraging given the overall aim of the INTEGRAL program to use these markers to improve short-term lung cancer risk assessment prior to LDCT screening^7,23,24,34^.

When evaluating the known mechanistic roles of the 36 proteins, we found that they have a wide range of molecular functions and include multiple growth factors (HGF, MK, IGFBP-1, IGFBP-2, TGF-alpha, VEGFA), tumor necrosis factor-receptors (TNFRSF6B, TNFRSF13B), and chemokines and cytokines (CXL17, GDF-15, OSM, SCF). SCF, the only protein that we found to be negatively associated with lung cancer, is involved in regulation of cell survival, proliferation and hematopoiesis^35^. The marker most strongly associated with lung cancer in our study—CEACAM5 (CEA)—had a stronger association for adenocarcinoma than for squamous cell carcinoma. CEACAM5 is a surface glycoprotein that is involved in cell adhesion, intracellular signaling, and tumor progression^36^. CEACAM5 is routinely used to monitor recurrence among colorectal cancer patients^37^, and was recently highlighted as a promising target for antibody-drug conjugate therapy of non-small cell lung cancer^38^.

When mapping the identified markers to the hallmarks of cancer, we found that the most frequently implicated hallmark was “activating invasion and metastasis” (19 markers), which was associated with proteins with known roles in the modulation of extracellular matrix during metastasis such as MMP12 and U-PAR^39,40^. The second most frequently implicated hallmark was “proliferative signaling”, which was associated with 17 markers, including growth factors such as HGF^41^, TGF-alpha^42^, and IGFBP-2^41^. Changes in proliferative signaling are common in lung tumors, as exemplified by the impact of deleterious mutations in well-described oncogenes, such as EGFR and KRAS^43^. The third most frequently implicated hallmark (14 proteins) was “tumor-promoting inflammation”, including markers such as CXCL9, CXCL13, CXL17, IL6, and IL2-RA. This highlights the central role for inflammation and the immune system in responding to or initiating the development of lung tumors^11,44^. Inflammation and metastasis in cancer are closely related^45^, as the invasion of vital organs by a tumor is regulated by matrix metalloproteases (MMP) and urinary plasminogen activator (UPA), both of which are regulated by NF-κB (regulator of a large array of genes involved in different processes of the immune and inflammatory responses)^45^. “Angiogenesis” was also associated with 14 proteins, including ANGPT2, CASP-8, and CEACAM5 which highlights the close relationship between invasion and metastasis and angiogenesis^46^.

To better understand the relationships between the 36 markers, we conducted a sparse graphical LASSO-based network analysis and observed specific associations between 12 proteins among lung cancer cases that did not appear among controls. These case-specific protein connections were clustered in three groups and were all broadly implicated in an extracellular defense response to somatic stress. In contrast, connections that were specific to controls appeared to be more strongly associated with a signaling response to cell proliferation. In seeking to establish a risk prediction model including multiple proteins, we would anticipate some redundancy in the risk discriminative performance of connected proteins. An interesting observation was that several of the proteins most strongly associated with lung cancer, including CEACAM5, IL6, and SCF, did not have any stable connections with the identified markers.

To understand why circulating concentrations of the identified proteins are associated with lung cancer diagnosis, and to assess whether they are likely to be specific to lung cancer—as opposed to cancer at other sites—we used publicly available expression data for a range of normal and tumor tissues. This analysis yielded two notable observations; first, that only three proteins, ALPP, SFTPA1, and MUC-16, were predominantly expressed in normal lung cells compared to cell types of other origins. In contrast, several proteins appeared to be primarily expressed by immune cells, although most were also expressed by other cell types. The second notable observation was that only one protein—SFTPA1—was predominantly expressed by lung tumor tissue compared to other tumor tissues, whereas most proteins were expressed in a wide range of cancer types. These complementary data suggest that few of the identified markers are likely to have originated in yet-to-be diagnosed lung tumor tissue, but rather are present in the circulation as a somatic response to subclinical cancer.

Associations between the identified markers and all-cause mortality after lung cancer diagnosis were weak. Three markers (U-PAR, CEACAM5, and CDCP1) were also weakly associated with all-cause mortality when measured as mRNA in lung tumor tissue in TCGA. Although these associations do not appear important, also considering that stage was not accounted for, they may be consistent with a role for some of the identified markers in tumor progression or an immune or inflammation response in lung tissue. For example, CDCP1 was previously associated with an increased risk of lung cancer in pre-diagnostic blood^28^, is overexpressed in lung cancer tissue^47^, and is associated with metastases and poor prognosis^47–50^. High U-PAR expression has been found associated with lower overall survival in patients with NSCLC^51^, and U-PAR is also studied as a therapeutic target in cancer^52^.

The key strength of our study is our large, rich data resource which was generated specifically to identify early detection markers of lung cancer. The study design, with pre-diagnostic samples drawn up to 3 years prior to clinical (not screen-detected) lung cancer diagnosis, ensured that identified markers were not influenced by the diagnosis itself or subsequent treatment, as in a retrospective case-control study of diagnosed cases^53^. By drawing samples from multiple studies, we were able to verify the consistency of associations across populations from the US, Europe, Southeast Asia, and Australia. Furthermore, our sample size provided 80% power to identify markers with an OR_sd_ of at least 1.26 after considering multiple testing, suggesting it is unlikely that we failed to identify any marker among the 1162 proteins that is of major use for early detection. Future discovery studies seeking to identify protein markers for early lung cancer detection may therefore consider using our results as an initial reference and focus additional investments on measuring non-overlapping sets of markers.

An important limitation of our study was that information on clinical stage was lacking for many cases. This limited our ability to comprehensively evaluate whether the identified markers were primarily driven by lung cancer diagnosed at late stage. However, based on the stage information available, we did not observe important differences between the OR estimates for early vs. late stage lung cancer.

Our controls were sampled directly from the same source population as cases and were individually matched to cases by detailed smoking characteristics, age, sex, and date of blood draw. This design protects against multiple types of bias that frequently affect biomarker studies. However, our nested case-control design does not readily allow us to establish absolute risk models, nor to evaluate the utility of our markers for risk prediction in the general population, because such metrics are strongly influenced by the highly selected controls. As described by Robbins et al.,^7^ we will address this question in a large, independent validation phase by analyzing pre-diagnostic blood samples from a larger sample of 1700 lung cancer cases and 2900 randomly selected cohort representatives including 10 additional cohorts participating in the Lung Cancer Cohort Consortium.

In future work, we plan to study the dynamics of the identified markers by evaluating repeat blood samples collected from the same individuals over time. As the majority of study participants in the cohorts were of European descent (except for the SCHS cohort which comprises mainly Han-Chinese participants), an important future aim is to determine whether any additional markers might be important specifically for populations of non-European ancestry. In addition, our study focused explicitly on people with a smoking history, and we consider it unlikely that the most relevant set of markers for lung cancer among people who never smoked were identified. Finally, we note that there is substantial scope for future studies to explore the potential biological roles of the identified markers in lung cancer development and progression.

To summarize, after screening 1162 proteins, we identified 36 markers of imminent lung cancer diagnosis with a wide range of functions and relevance across the hallmarks of cancer. Forthcoming studies will address the extent to which these markers can discriminate future lung cancer cases and their utility for early detection. Our study provides a potential view of the blood proteome in the years leading up to diagnosis of smoking-related lung cancer and can serve as a reference for investigations seeking to identify early protein markers of lung cancer.

## Methods

### Ethical approval

The protocol of the Lung Cancer Cohort Consortium (INTEGRAL project) was approved by the Ethics Committee of the International Agency for Research on Cancer (Project number 11–13). This study involved only secondary analysis of existing specimens and data. This research was performed in accordance with the Declaration of Helsinki.

### Study sample

A detailed justification for the study design and description of the study sample is available in Robbins et al.^7^. In brief, we included six prospective cohorts of diverse geographical origin amongst cohorts participating in LC3, all of which collected plasma or serum samples which were processed according to standard protocols and stored at −80C or in liquid nitrogen. These included the European Prospective Investigation into Cancer and Nutrition (EPIC)^54^ from several countries in Europe, The Northern Swedish Health and Disease Study (NSHDS)^55^ from Sweden, the Trøndelag Health Study (HUNT)^56^ from Norway, the American Cancer Society Cancer Prevention Study-II (CPS-II)^57^ from the US, the Melbourne Collaborative Cohort (MCCS)^58^ from Australia, and the Singapore Chinese Health Study (SCHS)^59^ from Singapore (descriptions of each cohort are provided in Robbins et al.^7^). Lung cancer cases were eligible if they reported a current or former history of daily cigarette smoking at recruitment and were diagnosed with a histologically confirmed lung cancer (C34) at most three years after blood draw. Controls were selected by incidence density sampling and matched 1:1 to cases based on age at blood draw (±1 year, relaxed to ±3 years for sets without available controls), date of blood draw (±1 month, relaxed to ±3 months), sex (self-reported), and cohort, as well as smoking status in four categories (people who formerly smoked and quit <10 or ≥10 years prior, and people who currently smoked <15 or ≥15 cigarettes per day). The final study sample included 731 lung cancer cases and 731 matched controls. All research participants provided written, informed consent, and the study was approved by the relevant Institutional Review Boards.

### Proteomic measurements

Circulating blood proteins were measured in plasma or serum using the Olink platform at Olink Proteomics (https://www.olink.com/) in Uppsala, Sweden. The Olink platform is based on proximity extension assays (PEA) that are highly sensitive, avoid cross-reactivity, and have high reproducibility^60^. Relative concentrations of up to 1162 unique proteins, distributed over 14 Olink panels, were measured by quantitative PCR (qPCR) (Supplementary Table 1). Measurements are expressed as normalized protein expression (NPX) values which are log-base-2 transformed. Details on quality control metrics and coefficients of variation are available in the Supplementary Methods and Supplementary Data 2a, b. Due to the high cost of Olink assays, we initially measured the complete available protein library only among the EPIC and NSHDS samples (*n* = 252 case-control pairs), and then assayed the HUNT, CPS-II, SCHS and MCCS samples (*n* = 479 case-control pairs) for a subset of promising panels which included between 392 and 484 proteins (see Robbins et al.^7^ and Supplementary Table 1). For proteins measured on multiple panels within a single cohort (*n* = 112 proteins with more than one measurement), we used the measurement with the highest variance and lowest missingness (see Supplementary Methods). Protein measurements were standardized within each cohort.

### Statistical analyses

The first step of our analysis aimed to identify proteins associated with imminent lung cancer diagnosis. Instead of using a single split-sample design, which can be subject to substantial influence from random chance, we applied a resampling-based algorithm which simulates a split-sample discovery and replication analysis repeated many times with many different random splits of the data. Specifically, in each of 500 iterations, we split the data into discovery (70%) and replication (30%) sets. In each of the 500 discovery and replication sets, we applied conditional logistic regression to estimate the odds ratio of lung cancer per standard deviation increment in relative concentration (log-base-2 transformed) of each protein [OR_sd_]. We applied this algorithm twice: once for the subset of 484 proteins measured in all six cohorts, and separately for the 678 proteins measured only in EPIC and NSHDS. In both algorithms, we balanced by cohort when splitting the data into random discovery (70%) and replication (30%) sets. In the algorithm including all 6 cohorts, we also ‘forced’ EPIC and NSHDS into the discovery set in every iteration since those data were used to choose the panels tested in the remaining four cohorts (Supplementary Methods, Supplementary Fig. 1). Additional details on how missing protein data were handled during the resampling algorithm are in the Supplementary Methods.

We considered proteins to show replicable associations with imminent lung cancer if, in at least 50% of iterations, the *p* value was below *p* < 0.05/effective-number-of-tests (ENT)^8^ in the discovery set and below 0.05 in the corresponding replication set. The ENT method accounts for multiple testing by applying a Bonferroni correction, but determines the number of independent tests as the number of principal components needed to explain 95% of the variance in protein abundance^8^.

As a sensitivity analysis, we assessed the difference between the results of our resampling approach and a standard, single split-sample design. Here, we included only EPIC and NSHDS in the discovery set, since these data were used to choose the panels measured in the other four cohorts, which were defined as the replication set. We identified proteins that had a false-discovery-rate (FDR)-adjusted *p* value below 0.05 in the discovery set and a *p* value below 0.05 in the replication set. We chose the less conservative FDR significance instead of ENT significance because the power in the discovery set for the single split-sample analysis was lower than in the resampling algorithm due to smaller sample size.

For the group of markers identified as associated with imminent lung cancer by the resampling algorithm, we carried out additional analyses using the full dataset. For each marker, we calculated odds ratios for lung cancer stratified by histological type, stage, smoking status, cohort, and lead time (time between blood draw and diagnosis) and examined trends by lead time (see Supplementary Methods). These stratified analyses did not account for multiple comparisons. To describe the association between each marker and smoking intensity, duration, and time since cessation, we used linear regression models fit among controls with adjustment for cohort, age, sex, and smoking status. Similar analysis was run in the full dataset (among cases and controls) while additionally adjusting for case-status. We also estimated stage at the time of blood draw for participants with available information on stage and histology using sojourn times specific to stage, sex, and histological type previously estimated by ten Haaf et al.^10^.

For the 36 identified proteins we ran pathway enrichment analysis using g:Profiler^16^ to examine the biological processes in which they are implicated, and we mapped these outcomes using Cytoscape version 3.9.1 with the EnrichmentMap and AutoAnnotate applications^61–63^. We then used the enrichment analysis results along with information available on GeneCards, the Human Protein Atlas, and Uniprot^13–15^ to match each protein’s function(s) to one or more of the Hallmarks of Cancer described by Hanahan and Weinberg^11,12^ in order to understand their biological roles within the development of cancer.

We also examined relationships between the identified markers. Separately among cases and controls, for pairs of proteins, we calculated Pearson’s correlation coefficients between the residuals of protein measurements after removing variance due to age, sex, and smoking status (‘residualized proteins’). To consider the relationships among all proteins simultaneously, we implemented sparse graphical network models. These models use a graphical LASSO-based resampling method on the partial correlations between residualized proteins to estimate a sparse set of connections among a set of proteins (see Supplementary Methods)^64^.

We subsequently evaluated the association between each identified marker and overall survival among participants with lung cancer, separately using circulating blood measurements and tumor gene expression. For blood measurements, we applied Cox proportional hazards regression based on the time from lung cancer diagnosis to death from any cause, with stratification of the baseline hazard by cohort and sex and adjustment for age at recruitment. Models also included an interaction between lead time and the protein measurement, so that the coefficient for the protein is interpretable as its effect at the time of lung cancer diagnosis. For tumor gene expression, we extracted lung tumor RNA-seq gene expression for 480 adenocarcinoma and 420 squamous cell lung cancer patients from The Cancer Genome Atlas (TCGA) (see Supplementary Methods).

We finally compared the cell-specific expression of the markers (mRNA expression) in tissue extracted from cancer-free individuals with expression in tumor tissue. Expression data were extracted from the Human Protein Atlas^65^ and the Pathology Atlas^66^. Details of these analyses are in the Supplementary Methods.

All statistical tests were two-sided, and all statistical analyses were performed using R version 4.1.2.

### Reporting summary

Further information on research design is available in the Nature Portfolio Reporting Summary linked to this article.

## Supplementary information

Supplementary Information

Reporting Summary

Description of Additional Supplementary Files

Supplementary Data

## Data Availability

The mission of the Lung Cancer Cohort Consortium (LC3) is to facilitate and carry out collaborative research on lung cancer risk and aetiology. The LC3 is committed to facilitating the use of LC3 data by the wider research community for research within its scientific mandate, including: 1- Research on the aetiology of lung cancer incidence and survival. 2- Research on lung cancer risk assessment, early detection, and screening. 3- Research on tobacco exposure and tobacco-related health outcomes. Access to the LC3 Data are restricted to researchers, who are affiliated with academic, non-profit, or governmental research institutions, and who have no links to the tobacco or arms industries. Access to LC3 Data cannot be granted to commercial entities and/or for commercial purposes, including development of patents. The LC3 Access Committee processes proposals to access LC3 data on a bi-monthly basis. Access to LC3 data can be obtained via the procedure outlined in the LC3 Access Policy which is available at the following link: https://www.iarc.who.int/wp-content/uploads/2021/12/LC3_Access_Policy.pdf. Other data sources: We also used publicly available mRNA expression from the Human Protein Atlas^65^ and the Pathology Atlas^66^. We also used lung tumor RNA-seq gene expression data from The Cancer Genome Atlas (TCGA)^67^ which is accessible upon request. Source data are provided with this paper.

## Source data

Source Data

## Code availability

Codes are available on github: GitHub- IET-IARC / LungCancerProteomics.
