# Supplementary material for: The blood proteome of imminent lung cancer diagnosis
Source: Nat Commun. 2023 Jun 1;14:3042. doi: 10.1038/s41467-023-37979-8 (PMC10235023; doi:10.1038/s41467-023-37979-8)
Supplement: Supplementary file 1 — Supplementary Information [file 41467_2023_37979_MOESM1_ESM.pdf]

The Blood Proteome of Imminent Lung Cancer Diagnosis

The Lung Cancer Cohort Consortium (LC3)

Contents

Supplementary Methods 2

Proteomics Measurements 2

Statistical Analyses 3

Protein Correlations and Network Analyses 4

All-Cause Mortality 5

Association of tumor gene expression with all-cause mortality among lung cancer cases 5

Tissue and Tumor Expression Proteins 5

Supplementary Data 6

All Supplementary Data are present in the same excel folder. Each data is present on a different sheet with the data number referenced in the manuscript. Sheets are ordered. 6

Supplementary Data 1: 6

Supplementary Data 2a and Supplementary Data 2b: 6

Supplementary Data 3: 6

Supplementary Data 4: 6

Supplementary Data 5: 6

Supplementary Data 6: 6

Supplementary Data 7: 6

Supplementary Data 8: 6

Supplementary Data 9: 6

Supplementary Data 10: 6

Supplementary Data 11: 6

Supplementary Data 12: 7

Supplementary Data 13: 7

Supplementary Tables: 8

Supplementary Table 1: Description of panels measured within cohorts........ 8

Supplementary Table 2: List of identified proteins implicated within each enriched pathway. 9

Supplementary Figures 10

Supplementary Fig. 1: Flow chart summarizing our method to identify protein markers associated with imminent lung cancer diagnosis. 10

Supplementary Fig. 2: Proportion of 500 random discovery-replication samples in which risk-associated proteins were replicated. 11
Supplementary Fig. 3: Depiction of proteins identified as being associated with risk of imminent lung cancer diagnosis using the main resampling algorithm and a standard split discovery-replication design. 12
Supplementary Fig. 4: Associations between the 36 identified and lung cancer risk, stratified by lung cancer stage (I/II vs III/IV). 13
Supplementary Fig. 5: Estimating lung cancer stage among cases at blood draw. 14
Supplementary Fig. 6: Heterogeneity in risk-associations for MMP12 and CEACAM5 by lung cancer histological type. 15
Supplementary Fig. 7: Heterogeneity in risk-associations for 11 proteins by lead time between blood draw and lung cancer diagnosis 16
Supplementary Fig. 8: Heterogeneity in risk-associations for VWA1 and IGFBP-1 by smoking status 17
Supplementary Fig. 9: Cross-sectional relationships between protein measurements and smoking exposure information. 18
Supplementary Fig. 10: Heterogeneity in risk-associations for S100A4 and RFNG between the Singapore Chinese Health Study (SCHS) vs. other cohorts from the USA, Europe, and Australia. 19
Supplementary Fig. 11: Pathway analysis considering the 36 identified markers. 20
Supplementary Fig. 12: Network of the enriched pathways with the 36 identified markers. 21
Supplementary Fig. 13: Correlation analyses among 36 proteins associated with imminent lung cancer identified among 731 cases and 731 matched controls in the Lung Cancer Cohort Consortium 22
Supplementary Fig. 14: Association between the 36 markers of imminent lung cancer diagnosis and overall mortality among lung cancer cases. 23
References 24

Supplementary Methods

Proteomics Measurements

Circulating proteins were measured using the Olink Proteomics multiplex platform (Uppsala, Sweden).[1] The technology is based on a proximity extension assay (PEA) technique that is highly sensitive and avoids cross-reactivity with high reproducibility. The full protocol of the PEA has been reported previously.[2] The proteins are allocated across 14 separate panels, each including 92 proteins focused on a specific area of disease or biology. All sample plates included four internal control samples to monitor the quality of assay performance, as well as the quality of individual samples. The quality control (QC) was performed in two steps:

1. The standard deviation of the internal controls was evaluated for each sample plate. Only data from sample plates with a standard deviation below 0.2 NPX were considered valid.

2. The quality of each sample was assessed by evaluating the deviation from the median value of the controls for each individual sample. Only samples that deviated less than 0.3

NPX from the median passed the quality control. Fewer than 5 samples failed this quality control check.

We initially used all panels to measure 1,160 unique proteins on samples from EPIC and NSHDS (n=252 case-control pairs, some proteins were measured on several panels and the total number assays was 1,290). As outlined by Robbins et al.,[3] because of the incremental cost implications of applying each additional Olink panel, we selected five to six panels (392- and 484 proteins) based on the results from EPIC and NSHDS data that were assayed on the remaining samples from HUNT, MCCS, CPS-II and SCHS (n=478 case-control pairs). Details on the panels measured for each cohort are available in Supplementary Table 1. The panels focus on proteins with relevance for different processes such as immunity (e.g. inflammation), cell regulation (e.g. regulation of cell proliferation, cell death/apoptosis), tissue generation and remodeling (e.g. angiogenesis, heart development), as well as mechanisms that are central to the initiation and progression of diseases such as cancer (e.g. angiogenesis, cell differentiation and adhesion) and neurology-related diseases. Pairs of case-control samples were plated together, with the pairs randomly allocated over 96-well plates. Protein concentrations were measured by quantitative PCR (qPCR) to quantify relative protein concentrations expressed as normalized protein expression (NPX) values on the log2 scale. Measurements below the lower limit of detection (LOD) were replaced with the LOD divided by the square-root of 2.[4]

Overall, 112 proteins were measured on more than one panel, with some proteins assayed on 5 different panels. For analysis, we chose one measurement per protein by cohort. First, for each protein we prioritized the measurements from the four panels measured on all cohorts (Cardiovascular III, Immuno-Oncology, Inflammation, and Oncology II). Then, if needed, we selected the measurement with the highest variance within each cohort. Protein measurements were standardized by cohort.

Statistical Analyses

Our resampling algorithm is described in the main text. Here, we provide additional details about its implementation.

To account for missing protein data (specifically when dealing with a protein that is not measured in a cohort), at each iteration for each protein we removed individuals with missing values for the protein in question before splitting the data into discovery and replication. Protein measures were rescaled at each iteration by cohort, separately for the discovery and replication set.

Because many proteins were only measured in the EPIC and NSHDS cohorts, to identify the markers associated with lung cancer, we applied the resampling algorithm twice: once on EPIC and NSHDS alone (n=678 proteins), and once on all cohorts together (n=392-484 proteins) (see Supplementary Figure 1). Case-sets were allocated randomly into discovery (70%) and replication (30%) while balancing by cohort. We calculated the effective number of tests separately for the two sets of proteins.

We carried out analyses to obtain preliminary estimates of the improvement in discrimination provided by each single protein beyond the PLCOm2012 risk model. For this analysis, we assigned missing values for COPD, family history of lung cancer and personal history of cancer as zeros (absence of the risk factor). Missing values for smoking intensity, duration, years since cessation, BMI and education were imputed by predictive mean

matching in multiple chained equations (MICE), stratified by cohort and smoking status.[3] To apply a consistent statistical approach, we first fit a logistic regression model to the logit of the PLCOm2012 score with adjustment for the matching factors (age, sex, year of blood draw and smoking status) and calculated the AUC for the model predictions. We note that these AUCs, calculated in data from matched cases and controls, are artificially reduced compared with AUCs that would be obtained in a representative population. Then, for each protein, we fit a logistic regression model including the protein and the logit of the PLCOm2012 risk score, also adjusted on matching factors. A separate model was fit with each protein alone, excluding the PLCOm2012 score. For each of these 3 models, we estimated the area under the ROC curve (AUC) based on the individual probabilities of lung cancer predicted by the model. We calculated the improvement in discrimination provided by each protein as the difference in AUCs between the model with the protein and the PLCOm2012 score versus the model with the PLCOm2012 score only. We used the pROC package in R to calculate the AUCs, found in Supplementary Data 4.

For the 36 proteins identified by the resampling algorithm, we assessed trends by pre-diagnostic lead time in the association between each protein and lung cancer risk. Here, we report the beta coefficient, p-value, and Z-score of the interaction between the protein and lead-time from a conditional logistic regression model additionally adjusted for the protein measurement.

Protein Correlations and Network Analyses

To evaluate how the identified proteins associate with each other distinctly among cases and controls, we first removed variation in protein concentrations due to age, sex, and smoking status by taking residuals from a linear regression of each protein (separately) on these three factors. We subsequently calculated Pearson's correlation coefficients among proteins, separately among cases and controls, and presented correlations with $p<0.05$.

To consider relationships between all markers associated with lung cancer simultaneously, we employed sparse graphical network models that estimate the network topology of protein correlations. We estimated these networks based on data from EPIC and NSHDS only to include all identified proteins. As for the pairwise correlation analysis, we applied the sparse graphical network models on the residuals from a linear regression of each protein on age, sex, and smoking status. We estimated the sparse network for the identified markers separately in cases and controls.

The sparse graphical network models use a graphical LASSO-based re-sampling method on the partial correlations between proteins to estimate a sparse set of connections between a set of proteins.[5] It has three main parameters: the LASSO penalization parameter (λ) to determine the degree of sparsity in the network, the threshold for the proportion of resamplings (π) where a given connection between two proteins is observed, and the per-family error rate (PFER) to determine a ceiling on the number of false protein-protein connections in a network. We set the PFER to < 5% of the total potential network size [$N\times(N-1)/2$]. Once the PFER is set, we chose values for λ and π by maximizing the negative log-likelihood estimator of the stability of the network. The resulting networks in cases and controls can be interpreted as the sparse and stable set of adjusted protein-protein connections, without direction.

We subsequently used these networks to identify the protein-protein connections that were common in control and case networks, that were unique to controls, and that were unique

to cases. We considered network statistics including normalized group-level centrality, as a measure of how structured the network of associations were around a central group of important proteins, calculated using the igraph package on R[6].

All-Cause Mortality

Association of tumor gene expression with all-cause mortality among lung cancer cases

For tumor gene expression analyses of the identified markers, we extracted lung tumor RNA-seq gene expression for 480 adenocarcinoma and 420 squamous cell lung cancer patients from The Cancer Genome Atlas (TCGA) project 2731 via dbGAP. We applied Cox regression to estimate hazard ratios for all-cause mortality based on a standard deviation increment in gene-expression. These models included stratification of the baseline hazard by sex and histological subtype and adjustment for age at diagnosis.

Tissue and Tumor Expression Proteins

Single cell mRNA expression data available through the Human Protein Atlas[7] was used to describe mRNA expression for genes that code for the identified markers taken from cancer-free individuals. Normalized expression levels were obtained using single cell RNA sequencing of 51 cell types from 13 different human tissues. Cell-specific expression was calculated as the ratio of each cell type expression to the total expression across all cell-types for each gene. We included epithelial, endocrine, endothelial, muscle, pigment, mesenchymal, and blood and immune cells. Expression in neuronal, glial, germ, trophoblast, and undifferentiated cells was not included because these tissues were unlikely to contribute significantly to circulating levels of these proteins in adult men and women. In Figure 4, individual cell type expression levels are shown for 4 lung-specific epithelial cell types, 7 blood and immune cell types, hepatocytes, endothelial cells, and pigment cells. Summed expression levels are shown for the 15 remaining epithelial cell types (labeled other epithelial cells), the 3 endocrine cell types, 3 mesenchymal cell types, and 2 muscle cell types. Expression was defined to be minimal for cell types with <5% of the total mRNA expressed.

The same methodology was applied to mRNA expression data from the Pathology Atlas[8] to quantify expression by various tumor types.

Supplementary Data

All Supplementary Data are present in the same excel folder. Each data is present on a different sheet with the data number referenced in the manuscript. Sheets are ordered.

Supplementary Data 1: Characteristics of 731 lung cancer cases and 731 matched controls stratified by cohort.

Supplementary Data 2a and Supplementary Data 2b: Quality controls of assay measures in the EPIC and NSHDS cohorts (1a), and in in the CPS, HUNT, MCCS and SCHS cohorts (1b).

Footnote: Follow-up time for lung cancer may be shorter than follow-up time for mortality due to different end dates for the completeness of cancer registry vs mortality registry data.

Supplementary Data 3: Proportion of samples below the LOD.

Supplementary Data 4: Observed effect size of all measured proteins with lung cancer risk in the full data.

Supplementary Data 5: Proportion of 500 random discovery-replication samples in which risk-associated proteins were replicated.

Supplementary Data 6: Comparison of the estimated associations between each protein and lung cancer risk identified by the single split design vs the resampling algorithm.

Supplementary Data 7: Stratified associations of the 36 identified markers with lung cancer risk & AUCs across stage strata.

Supplementary Data 8: Stratified associations of the 36 identified markers with lung cancer risk across different strata.

Supplementary Data 9: Trends by lead time in the association between the 36 identified markers and lung cancer risk.

Supplementary Data 10: Lung cancer odds ratios for the 36 proteins associated with imminent lung cancer before and after detailed adjustment for smoking intensity and duration.

Footnote: we defined lead time as the time (in years) elapsed between blood draw and clinical diagnosis of lung cancer.

Supplementary Data 11: Centralities of the penalized networks of the 36 identified markers.

Footnote: Degree centrality represents the number of edges each node has (i.e. the number of proteins each protein is directly connected to).

Betweenness centrality represents the importance of each node to the flow of the network by assessing the number of short paths between two nodes each node is on (i.e. if protein A is connecting protein B and C and there's no other link to B and C, then A would have a high betweenness centrality. If protein D is connected to B and has no other connection, D is therefore not linking any proteins and will have a low betweenness centrality).

Closeness centrality represents the average distance between each node and the other nodes (i.e. for each protein we calculate the inverse of the sum of the distances to every other protein). The higher the closeness is, the more each protein is efficiently related to the other proteins in the network.

Eigen vector centrality is an extension of degree centrality. It adjusts the centrality degree assigned by the number of direct links to each protein for their “power”. In other words, if protein A and protein B are each independently linked to 5 other proteins then both have a high degree centrality. However if the 5 proteins linked to protein A have no importance in the network (are not linked to other proteins) while the 5 proteins linked to B are linked to other proteins in the network than protein B will have a higher eigenvector centrality than protein A.

Supplementary Data 12: Association between the 36 markers of imminent lung cancer diagnosis and overall mortality among lung cancer cases, based on direct measurements among participants in the Lung Cancer Cohort Consortium and tumor gene expression in TCGA.

Supplementary Data 13: Vital status outcomes among lung cancer cases.

Supplementary Tables:

Supplementary Table 1: Description of panels measured within cohorts (*Robbins et al, Ann Epidemiol., 2022,* https://doi.org/10.1016/j.annepidem.2022.10.014)

	Full Discovery		Targeted Discovery			
Cohorts	EPIC	NSHDS	SCHS	CPS-II	HUNT	MCCS
Number of cases	188	64	92	115	163	108
Number of panels measured	13	13	5	6	5	6
Number of Olink IDs*	1196	1196	460	552	460	552
Number of unique proteins*	1161	1161	394	484	392	484
Proteomics Panels:						
Cardiovascular III	X	X	X	X	X	X
Inflammation	X	X	X	X	X	X
Immuno-Oncology	(X)	(X)	X	X	X	X
Oncology II	X	X	X	X	X	X
Oncology III	X	X	X	X		X
NeuroExploratory	X	X		X	X	X
Cardiometabolic	X	X				
Cardiovascular II	X	X				
Cell Regulation	X	X				
Development	X	X				
Immune Response	X	X				
Metabolism	X	X				
Neurology	X	X				
Organ Damage	X	X				

Selection of panels measured in the replication phase (SCHS, MCCS, CPS-II and HUNT) was based on the the number of highly ranked and consistently selected proteins in EPIC and NSHDS.

*Some proteins are measured on multiple panels and therefore have multiple Olink IDs for the same protein. In these cases, for each protein, we chose a single Olink ID for analysis by choosing the one that was measured on more cohorts, and then if needed, the Olink ID with the highest variance.

(X): all the proteins from the Immuno-Oncology panel are included on other panels assayed as indicated.

Overall, 1163 unique proteins were measured by Olink. 1161 is the number of unique proteins measured in EPIC and NSHDS (2 proteins from the NeuroExploratory panel (ADGRB3 and LTBP3) were not measured in EPIC and NSHDS but had measurements in the remaining cohorts.
Throughout the manuscript we refer to 1162 proteins analyzed, as 1 protein (MAPT) was excluded from the analysis due to invariant measurements (standard deviation = 0) in all cohorts, and refer to n=1160 proteins analyzed in EPIC and NSHDS.
EPIC: Investigation into Cancer and Nutrition, NSHDS: The Northern Swedish Health and Disease Study (NSHDS), HUNT: the Trøndelag Health Study, CPS-II: the American Cancer Society Cancer Prevention Study-II, MCCS: the Melbourne Collaborative Cohort, SHCS: Singapore Chinese Health Study (SCHS)

Supplementary Table 2: List of identified proteins implicated within each enriched pathway.

Pathway name	Proteins included
Phosphorylation cascades (mapk)	CHI3L1; CXL17; GDF15; HGF; IL2RA; IL6; SCF; OSM; U-PAR; EN-RAGE; TGFA
Response to chemical, organic and cytokine stimuli	ANGPT2; CASP8; CHI3L1; CXCL13; CXL17; CXCL9; GDF15; HGF; IFI30; IGFBP1; IGFBP2; IL2RA; IL6; LAMP3; MK; MMP12; OSM; U-PAR; EN-RAGE; SYND1; SPINT1; TGFA; TNFRSF6B; TNFSF13B; VEGFA
Response to wounding	IGFBP1; IL6; MK; MMP12; U-PAR; SYND1; TFPI2; TGFA
Regulation of cellular component	CASP8; CFHR5; CHI3L1; CXCL13; CXL17; CXCL9; GDF15; HGF; IGFBP1; IGFBP2; IL2RA; IL6; SCF; MK; MMP12; OSM; U-PAR; S100A11; EN-RAGE; SYND1; SPINT1; TGFA; TNFSF13B; VEGFA
Regulation of developmental processes	ANGPT2; CASP8; CEACAM5; CHI3L1; CXCL13; CXL17; CXCL9; GDF15; HGF; IL2RA; IL6; SCF; MK; MMP12; OSM; U-PAR; SPINT1; TNFSF13B; VEGFA
Multicellular organismal production	ANGPT2; CASP8; CHI3L1; CXL17; HGF; IL2RA; IL6; SCF; MK; MMP12; OSM; SPINT1; VEGFA
Intracellular signal transduction	ANGPT2; CASP8; CFHR5; CHI3L1; CXCL13; CXL17; CXCL9; GDF15; HGF; IFI30 ; IGFBP1; IGFBP2; IL2RA; IL6; SCF; LAMP3; MK; MMP12; MUC16; OSM; U-PAR; S100A11; EN-RAGE; SYND1; SFTPA1; SPINT1; TFPI2; TGFA; TNFRSF6B; TNFSF13B; VEGFA; VWA1
Immune system processes	ANGPT2; CASP8; CEACAM5;CFHR5; CHI3L1; CXCL13; CXL17; CXCL9; IFI30; IGFBP2; IL2RA; IL6; SCF; LAMP3; MK; MMP12; MUC16; OSM; U-PAR; S100A11; EN-RAGE; SYND1; SFTPA1; TNFSF13B; VEGFA
Regulation of biological processes	ANGPT2; CASP8; CEACAM5; CXCL13; CXL17; CXCL9; GDF15; HGF; IGFBP1; IGFBP2; IL2RA; IL6; SCF; LAMP3; MK; MMP12; OSM; U-PAR; S100A11; SPINT1; TFPI2; TGFA; TNFRSF6B; VEGFA; WFDC2
Cell death	CASP8; CEACAM5; CHI3L1; HGF; IL2RA; IL6; SCF; LAMP3; MK; U-PAR; TGFA; TNFRSF6B; VEGFA
Defense and inflammatory response	ANGPT2; CASP8; CFHR5; CHI3L1; CXCL13; CXL17; CXCL9; HGF; IFI30; IGFBP1; IL2RA; IL6; MK; MMP12; MUC16; OSM; U-PAR; EN-RAGE; SYND1; SFTPA1; TFPI2; TGFA; VEGFA; VWA1
Response to external stimulus	ANGPT2; CASP8; CFHR5; CHI3L1; CXCL13; CXL17; CXCL9; GDF15; HGF; IFI30; IGFBP2; IL2RA; IL6; MK; MMP12; MUC16; OSM; U-PAR; EN-RAGE; SFTPA1; VEGFA
Cell mobility	ANGPT2; CEACAM5; CXCL13; CXL17; CXCL9; HGF; IL6; SCF; MK; MMP12; U-PAR; S100A11; EN-RAGE; SYND1; VEGFA
Receptor ligand activity	ANGPT2; CASP8; CXCL13; CXCL9; GDF15; HGF; IGFBP1; IGFBP2; IL6; SCF; MK; OSM; U-PAR; EN-RAGE; SPINT1; TFPI2; TGFA; TNFSF13B; VEGFA; WFDC2
Cell adhesion	ANGPT2; CEACAM5; CXCL13; IGFBP2; IL2RA; IL6; SCF; MK; MMP12; MUC16; U-PAR; S100A11; TNFSF13B; VEGFA
Lymphocyte regulation	IGFBP2; IL2RA; IL6; TNFSF13B
Angiogenesis and blood structure development	ANGPT2; CHI3L1; CXCL13; CXL17; CXCL9; GDF15; HGF; IL6; MK; MMP12; SYND1; SPINT1; TGFA; TNFSF13B; VEGFA; VWA1
Differentiation pathway	HGF; IL6; SCF; VEGFA
Cell proliferation	CXCL9; HGF; IGFBP2; IL2RA; IL6; SCF; MK; MMP12; OSM; S100A11; SPINT1; TGFA; TNFSF13B; VEGFA
Peptidase& endopeptidase activity	CASP8; HGF; LAMP3; U-PAR; SPINT1; TFPI2; VEGFA; WFDC2
Cytokine receptor binding	CASP8; CXCL13; CXCL9; IL6; SCF; OSM; TNFSF13B; VEGFA
Signaling immune system	CASP8; CFHR5; CHI3L1; HGF; IFI30; IL2RA; IL6; MUC16; OSM; U-PAR; S100A11; EN-RAGE; SYND1; SFTPA1; TGFA; TNFRSF6B; TNFSF13B; VEGFA
Cell periphery	ALPP; ANGPT2; CASP8; CDCP1; CEACAM5; CXCL9; IGFBP2; IL2RA; IL6; SCF; LAMP3; MUC16; U-PAR; EN-RAGE; SYND1; SPINT1; TGFA; TNFSF13B
Extracellular space	CHI3L1; GDF15; MK; MMP12; TFPI2; VEGFA; VWA1
Cell surface	ALPP; CEACAM5; CXCL9; IL2RA; U-PAR; SYND1; TGFA; VEGFA
Extracellular matrix	CHI3L1; GDF15; MK; MMP12; TFPI2; VEGFA; VWA1
Lung fibrosis	HGF; IL6; SFTPA1; TGFA
Peptidyl tyrosine phosphorylation	HGF; IL6; SCF; OSM; TGFA; VEGFA
Pi3k akt signaling	ANGPT2; HGF; IL2RA; IL6; SCF; OSM; TGFA; VEGFA
Allograft rejection	CASP8; CXCL13; CXCL9; IL2RA; VEGFA
Induced photodynamic therapy	ANGPT2; IGFBP1; IGFBP2; TGFA; VEGFA

Supplementary Figures

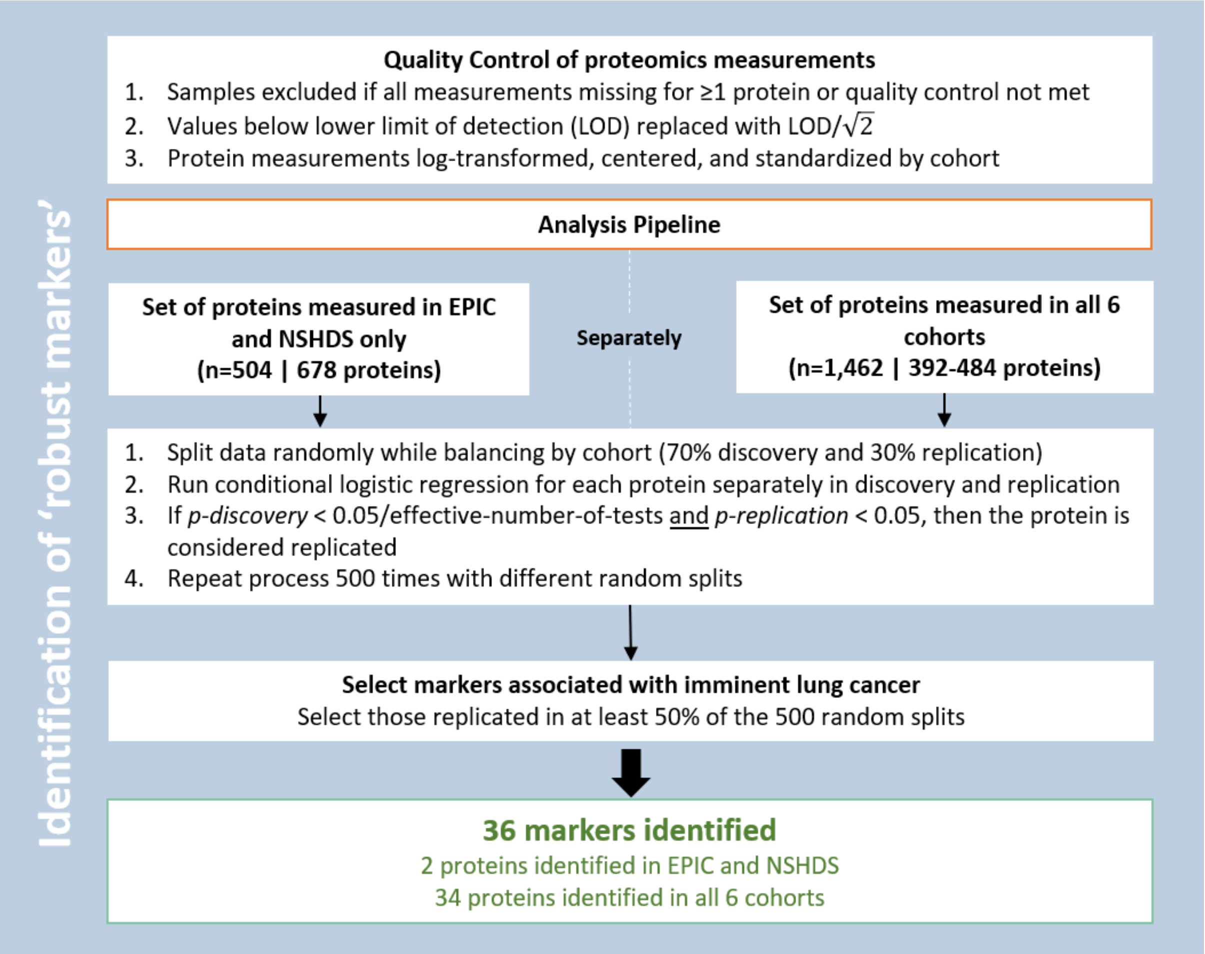

Supplementary Fig. 1: Flow chart summarizing our method to identify protein markers associated with imminent lung cancer diagnosis.

EPIC: The European Prospective Investigation into Cancer and Nutrition; NSHDS: Northern Sweden Health and Disease Study.
HUNT: The Trøndelag Health Study; MCCS: The Melbourne Collaborative Cohort Study; SCHS: The Singapore Chinese Health Study; CPS-II: The Cancer Prevention Study II.

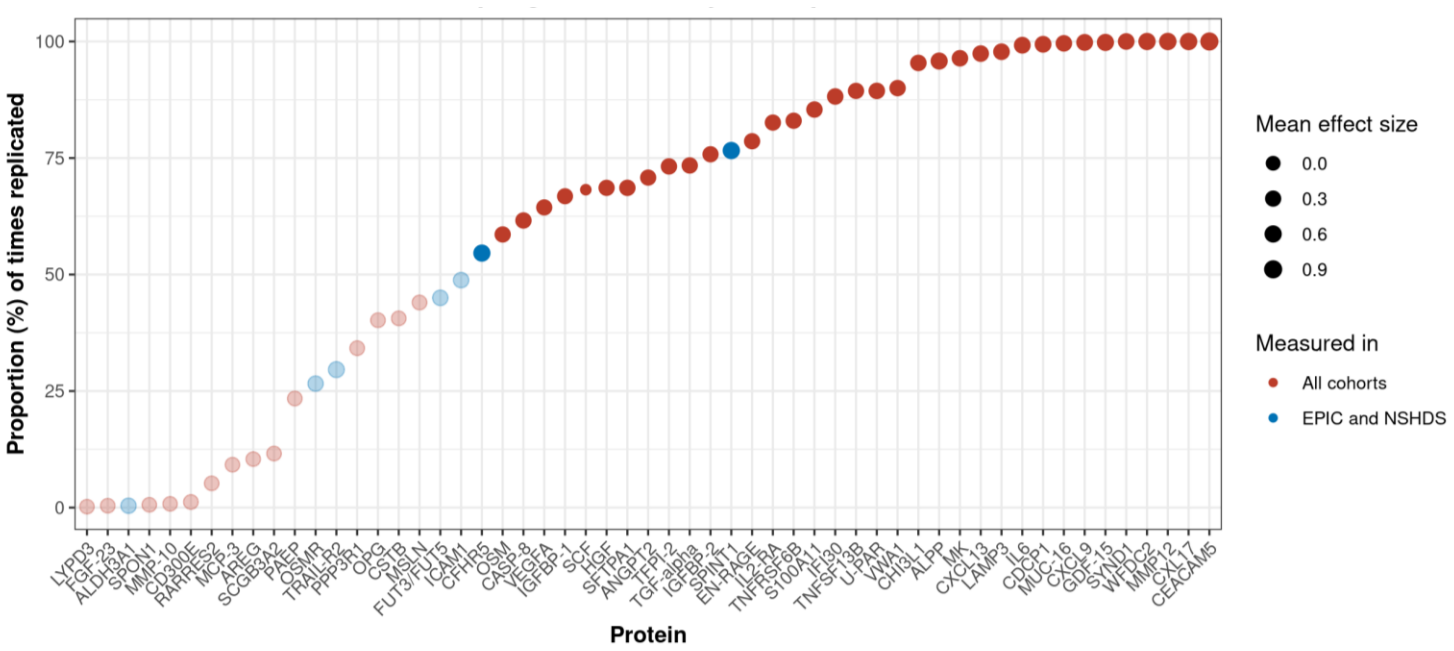

Supplementary Fig. 2: Proportion of 500 random discovery-replication samples in which risk-associated proteins were replicated.

We defined replicated biomarkers as biomarkers with an association below p<0.05/ENT in the discovery set and p<0.05 in replication set in one iteration. We calculated the mean effect size using beta estimates in training sets of the iterations where the protein was replicated. The number of times each protein was selected can be found in Supplementary Data 5. Source data are provided as a Source Data file.

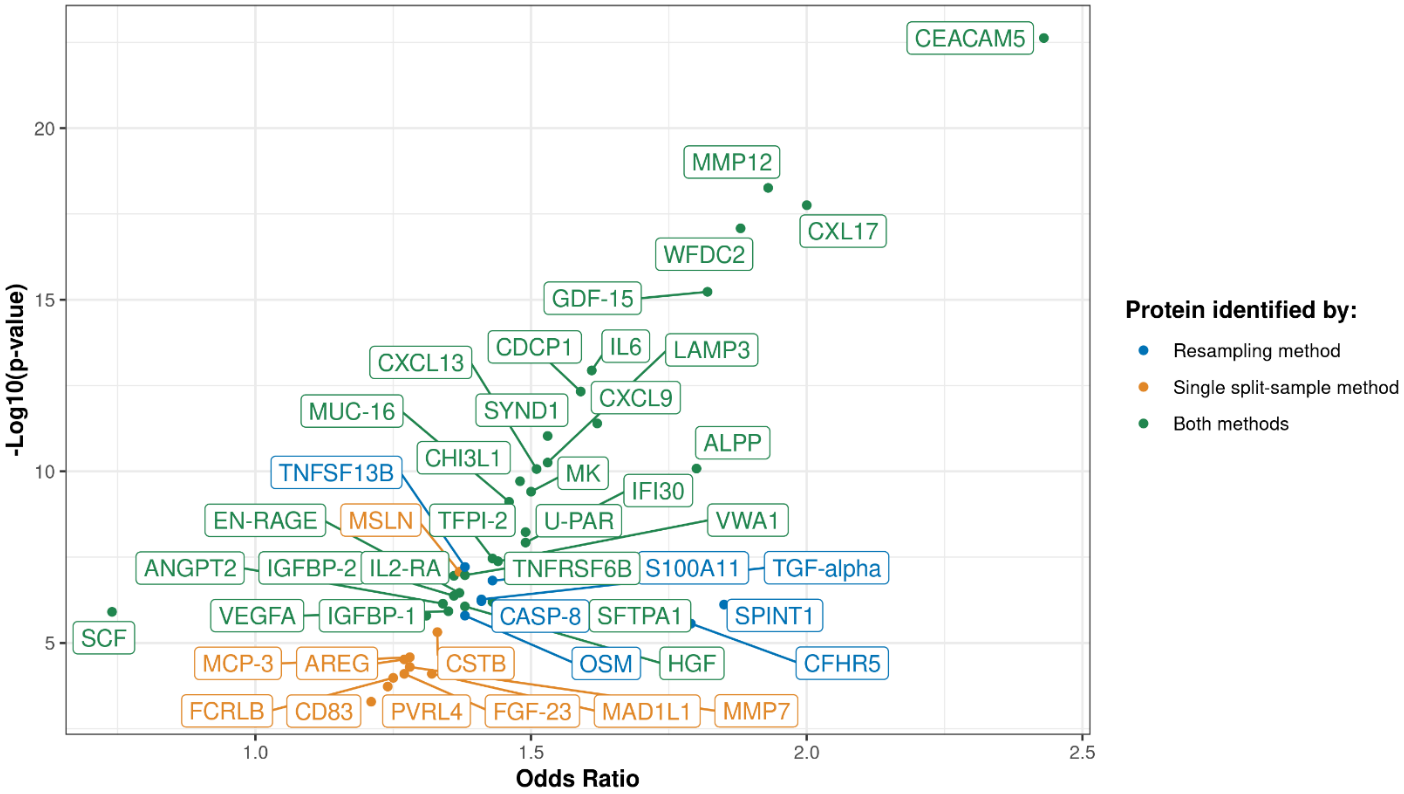

Supplementary Fig. 3: Depiction of proteins identified as being associated with risk of imminent lung cancer diagnosis using the main resampling algorithm and a standard split discovery-replication design.

The volcano plot depicts the odds ratios (x-axis) and pvalues of the association of each protein with lung cancer risk in the full datasets. Proteins are colored depending on the method by which they are identified. Proteins identified by the resampling method are the proteins referred to in the main manuscript; they had p<0.05/ENT in the discovery set (random 70% of the data) and p<0.05 in replication set (random 30% of the data) in at least 250 iterations out of 500 performed. The proteins identified using the single split-sample method were the proteins that had an FDR adjusted p<0.05 in the discovery set as defined by the design of the INTEGRAL Program (EPIC and NSHDS) and had p<0.05 in the replication set (MCCS, SCHS, CPS-II, HUNT). Odds ratios and p-values for the association of each protein in the discovery and replication sets of the single split methods are found in Supplementary Data 6. Source data are provided as a Source Data file.

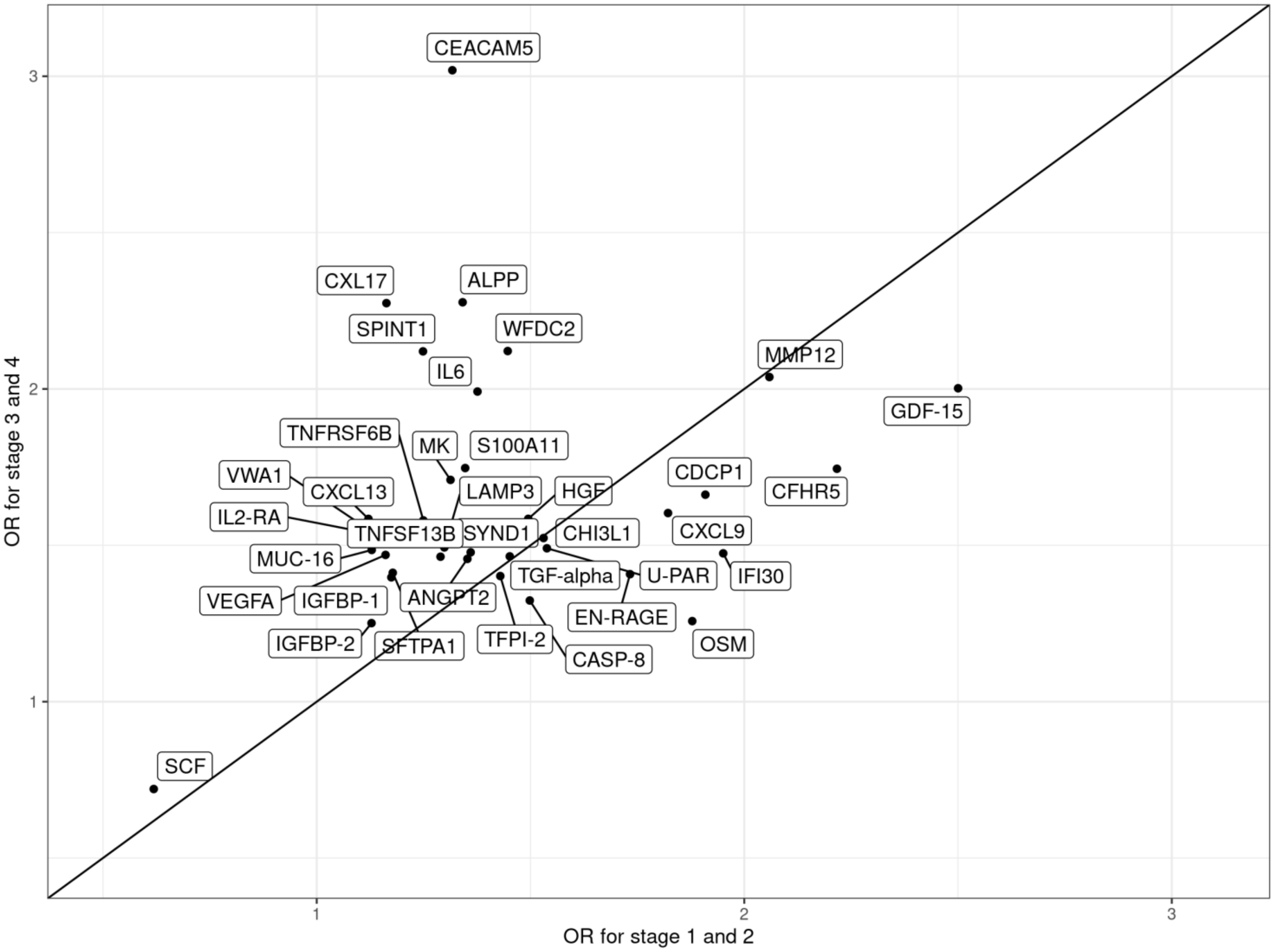

Supplementary Fig. 4: Associations between the 36 identified and lung cancer risk, stratified by lung cancer stage (I/II vs III/IV).

The x-axis shows the odds ratios (ORs) of each protein's association with lung cancer risk estimated among individuals diagnosed with early stage lung cancer (stage 1-2); the y-axis represents ORs estimated among individuals diagnosed with late stage lung cancer (stage 3-4). Source data are provided as a Source Data file.

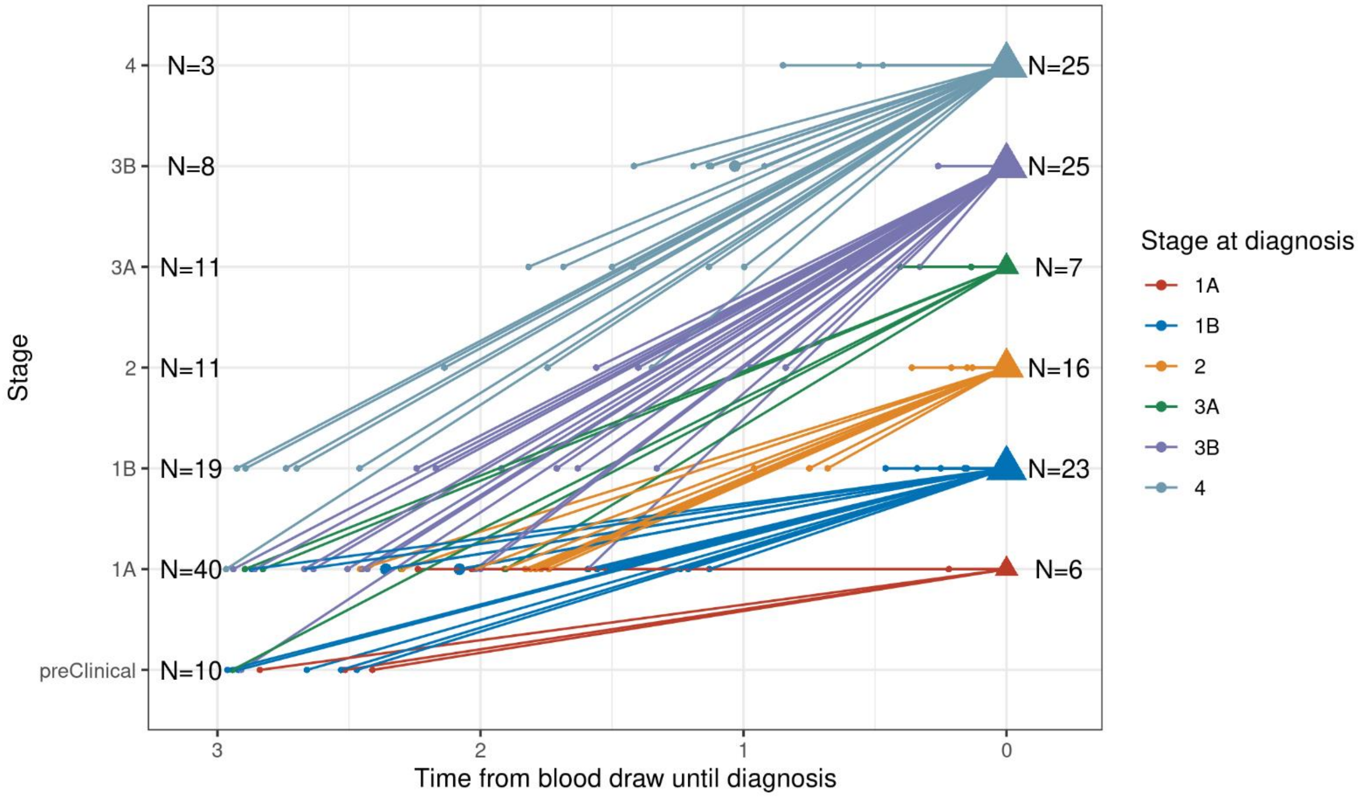

Supplementary Fig. 5: Estimating lung cancer stage among cases at blood draw.

The Y-axis represents the stage of lung cancer at different times (at blood draw vs at diagnosis t=0, represented by the X-axis). The stages at blood draw were estimated by sex, histology and time from blood-draw until diagnosis according to ten Haaf et al., 2015, CEBP. These analyses were done on 102 cases only with detailed and complete information on stage and histology. Source data are provided as a Source Data file.

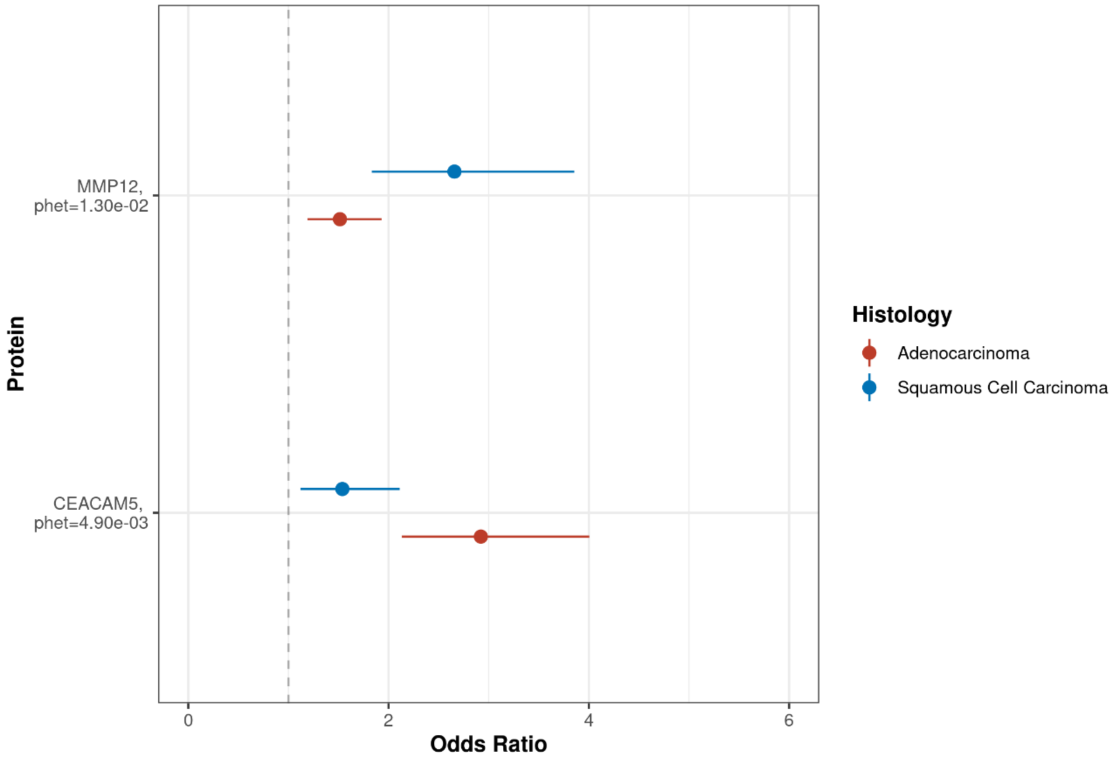

Supplementary Fig. 6: Heterogeneity in risk-associations for MMP12 and CEACAM5 by lung cancer histological type.

Odds ratios (ORs) of proteins with different effects on lung cancer risk by histology are presented (adenocarcinoma vs squamous cell carcinoma, phet <0.05). Data for 95% confidence intervals is presented as $e^{(\beta \pm 1.96 \times sd)}$. β is the estimate (log (OR)) from each conditional logistic regression, and sd is their respective standard deviation. Number of samples used are presented in Supplementary Data 8. Source data are provided as a Source Data file.

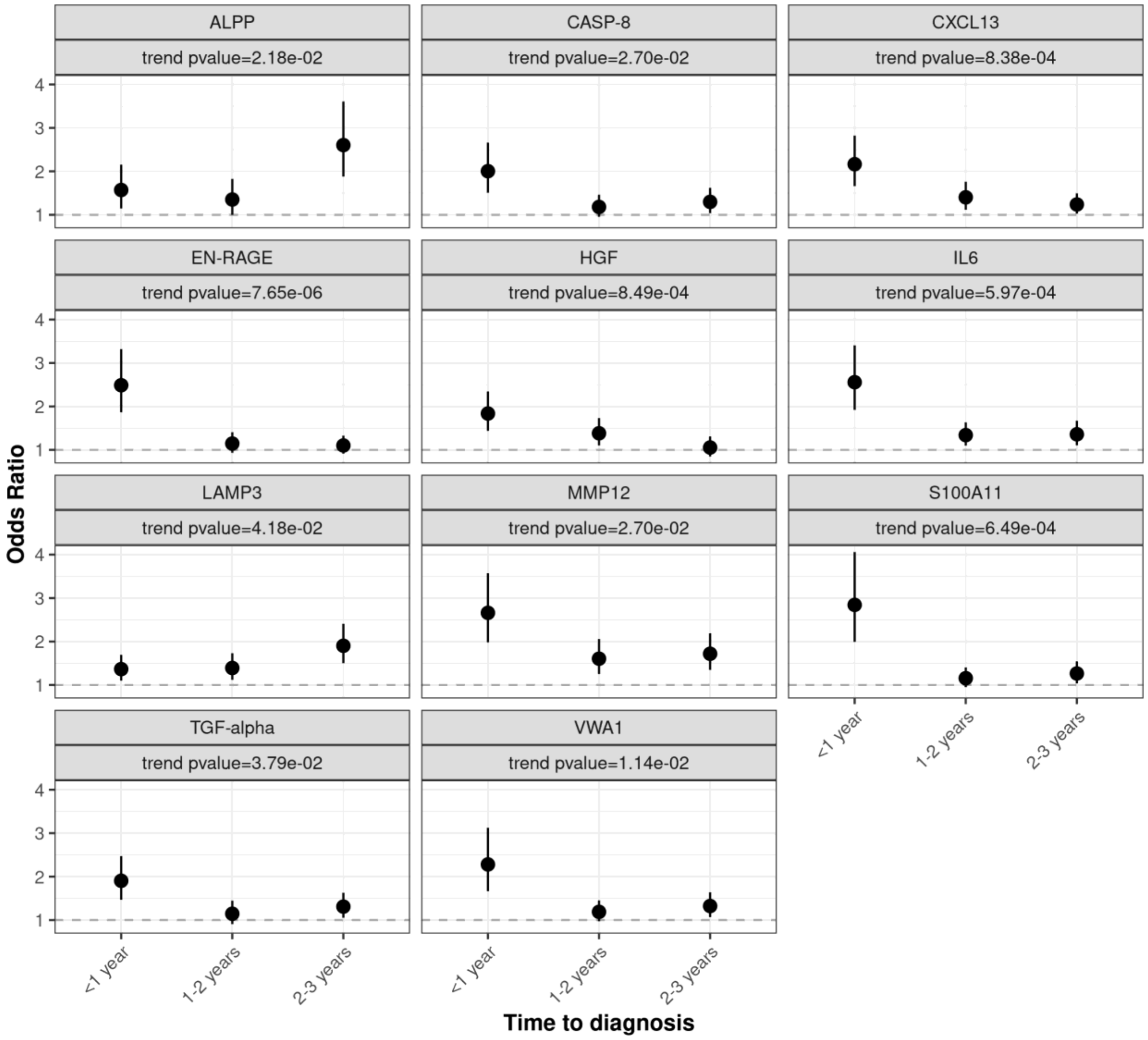

Supplementary Fig. 7: Heterogeneity in risk-associations for 11 proteins by lead time between blood draw and lung cancer diagnosis.

Odds ratios (ORs) of proteins with different effects on lung cancer risk by lead-time are presented (ptrend <0.05). Data for 95% confidence intervals is presented as $e^{(\beta \pm 1.96 \times sd)}$. β is the estimate ($\log(OR)$) from each conditional logistic regression, and sd is their respective standard deviation. Number of samples used are presented in Supplementary Data 8. Source data are provided as a Source Data file.

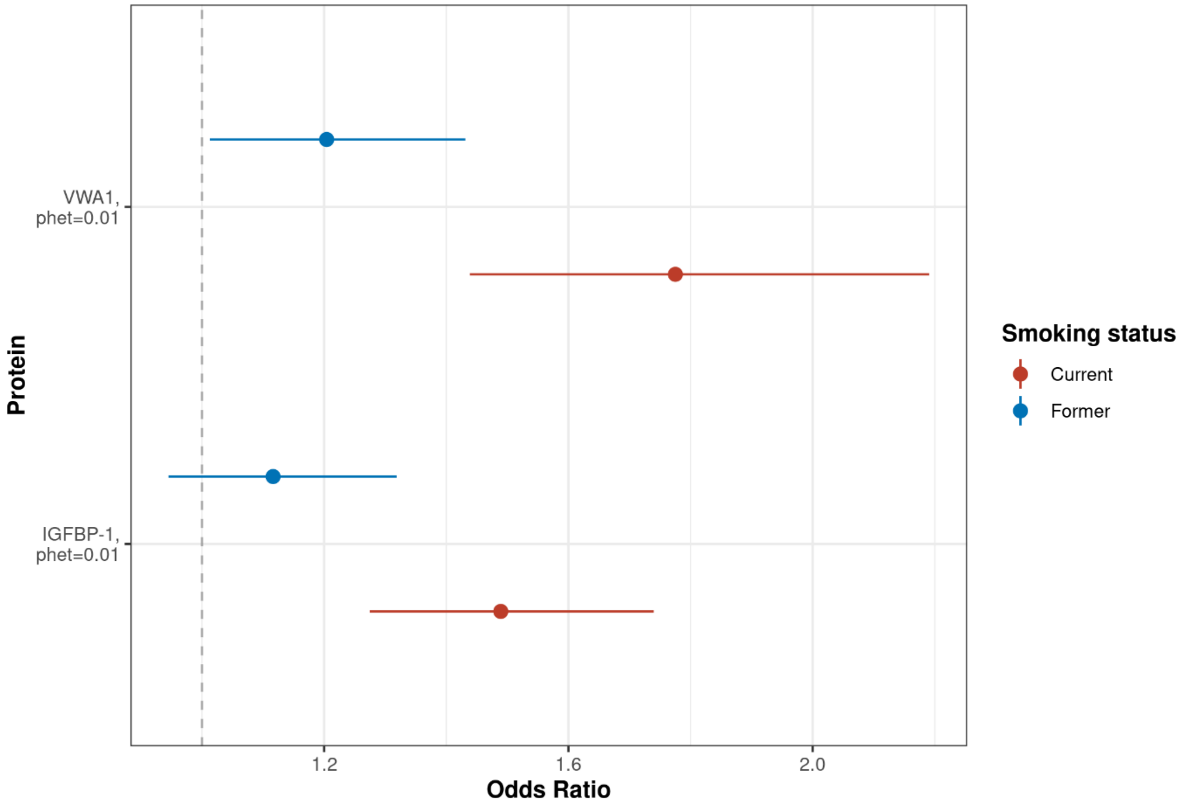

Supplementary Fig. 8: Heterogeneity in risk-associations for VWA1 and IGFBP-1 by smoking status.

Odds ratios (ORs) of proteins with different effects on lung cancer risk by smoking status are presented (current vs former smokers, phet <0.05). Data for 95% confidence intervals is presented as $e^{(\beta \pm 1.96 \times sd)}$. β is the estimate ($\log(OR)$) from each conditional logistic regression, and sd is their respective standard deviation. Number of samples used are presented in Supplementary Data 8. Source data are provided as a Source Data file.

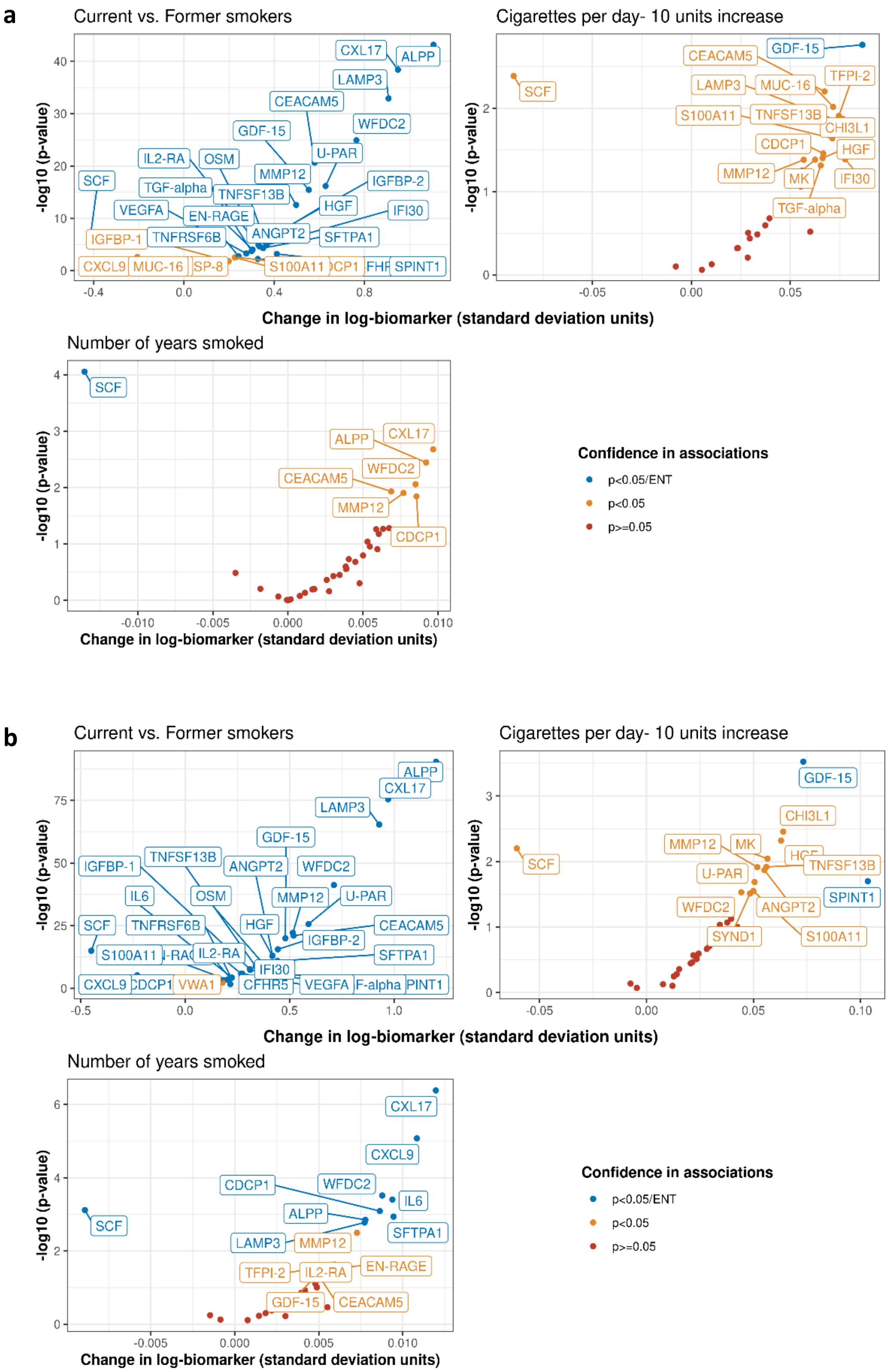

Supplementary Fig. 9: Cross-sectional relationships between protein measurements and smoking exposure information. The beta estimates from linear regression of each protein (outcome) against each risk factor are shown after adjusting for age, sex, smoking status (former vs current) and cohort, **a** among controls, **b** among all participants while further adjusting on case status. Source data are provided as a Source Data file.

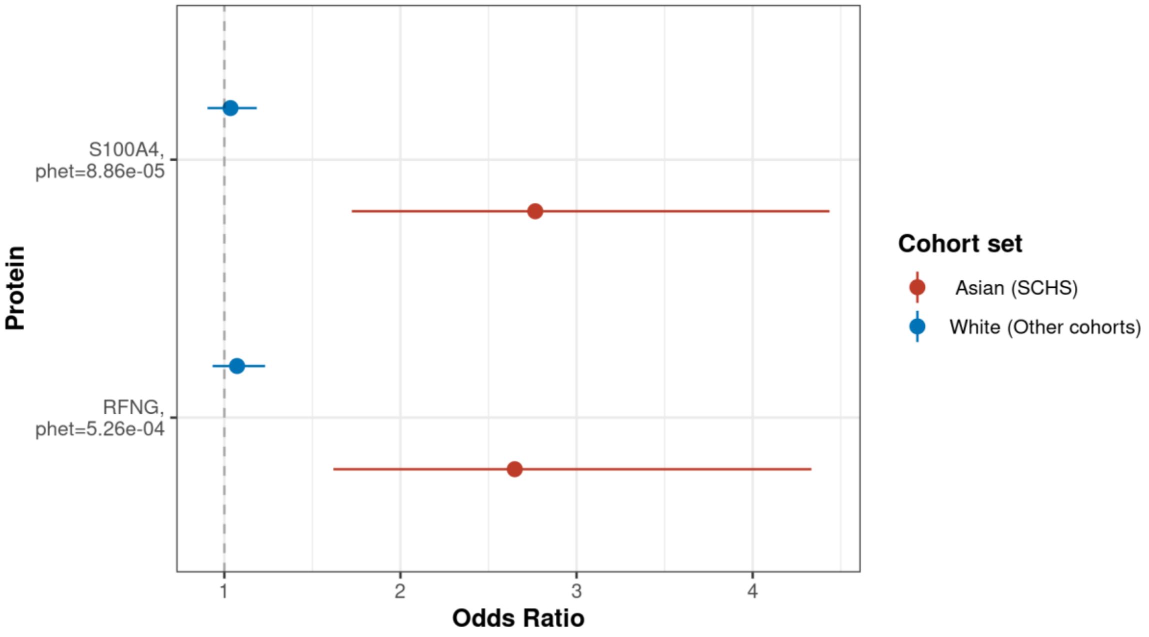

Supplementary Fig. 10: Heterogeneity in risk-associations for S100A4 and RFNG between the Singapore Chinese Health Study (SCHS) vs. other cohorts from the USA, Europe, and Australia.

Odds ratios (ORs) of proteins with different effects (phet <0.05) on lung cancer risk between Asian study participants (SCHS cohort) and non-Asian study participants (all other cohorts). The proteins were identified after estimating the effects of all proteins on lung cancer risk in the SCHS cohort alone. S100A4 and RFNG were associated with lung cancer risk in SCHS (pvalue<0.05/ENT) but were not among the 36 risk proteins identified in the overall study sample. Data for 95% confidence intervals is presented as $e^{(\beta \pm 1.96 \times sd)}$. β is the estimate ($\log(OR)$) from each conditional logistic regression, and sd is their respective standard deviation. Source data are provided as a Source Data file.

id	source	term_id	term_name	term_size	intersection_size		p_value
1	GO:CC	GO:0005576	extracellular region	4302	32		1.8e-16
2	GO:BP	GO:0050896	response to stimulus	9000	29		2.3e-03
3	GO:CC	GO:0005615	extracellular space	3368	27		7.1e-13
4	GO:BP	GO:0048519	negative regulation of biological process	5918	26		3.4e-05
5	GO:BP	GO:0048518	positive regulation of biological process	6309	26		1.4e-04
6	GO:BP	GO:0051716	cellular response to stimulus	7494	25		3.0e-02
7	GO:CC	GO:0071944	cell periphery	6270	25		1.3e-04
8	GO:BP	GO:0042221	response to chemical	4383	23		2.1e-05
9	GO:BP	GO:0048523	negative regulation of cellular process	4749	23		1.0e-04
10	GO:BP	GO:0048522	positive regulation of cellular process	5641	23		2.9e-03
11	GO:BP	GO:0007165	signal transduction	5993	23		9.2e-03
12	GO:BP	GO:0032502	developmental process	6424	23		3.3e-02
13	GO:BP	GO:0023052	signaling	6492	23		4.0e-02
14	GO:BP	GO:0007154	cell communication	6551	23		4.7e-02
15	GO:BP	GO:0006950	response to stress	3938	21		1.3e-04
16	GO:BP	GO:0048583	regulation of response to stimulus	3970	21		1.5e-04
17	GO:BP	GO:0009605	response to external stimulus	2814	19		2.0e-05
18	GO:BP	GO:0007166	cell surface receptor signaling pathway	2816	19		2.0e-05
19	GO:BP	GO:0002376	immune system process	2842	19		2.3e-05
20	GO:BP	GO:0010033	response to organic substance	3034	19		7.0e-05
21	GO:BP	GO:0070887	cellular response to chemical stimulus	3049	19		7.6e-05
22	GO:BP	GO:0048584	positive regulation of response to stimulus	2214	17		2.8e-05
23	GO:CC	GO:0031982	vesicle	3973	17		1.7e-02
24	GO:MF	GO:0005102	signaling receptor binding	1555	17		5.0e-08
25	REAC	REAC:R-HSA-168256	Immune System	2039	17		1.2e-03
26	GO:BP	GO:0071310	cellular response to organic substance	2405	16		6.9e-04
27	GO:BP	GO:0050793	regulation of developmental process	2499	16		1.2e-03
28	GO:BP	GO:0009653	anatomical structure morphogenesis	2722	16		3.7e-03
29	GO:BP	GO:0051239	regulation of multicellular organismal process	2753	16		4.4e-03
30	GO:BP	GO:0009966	regulation of signal transduction	2968	16		1.2e-02

g:Profiler (biit.cs.ut.ee/gprofiler)

Supplementary Fig. 11: Pathway analysis considering the 36 identified markers.

Pathway enrichment analysis with g:Profiler. The column called source represents the database used to extract the information. Term_id represents the ID that can be used to find information on the identified pathway in the different ontology databases. Term name is the name given to the identified pathway. Term size is the number of proteins that are attributed to the identified pathway. Intersection size is the number of proteins (from a given list, in our case from the 36 identified proteins) that are found in the pathway. P-value is the p-value for the enrichment of the 36 proteins within each pathway**.**

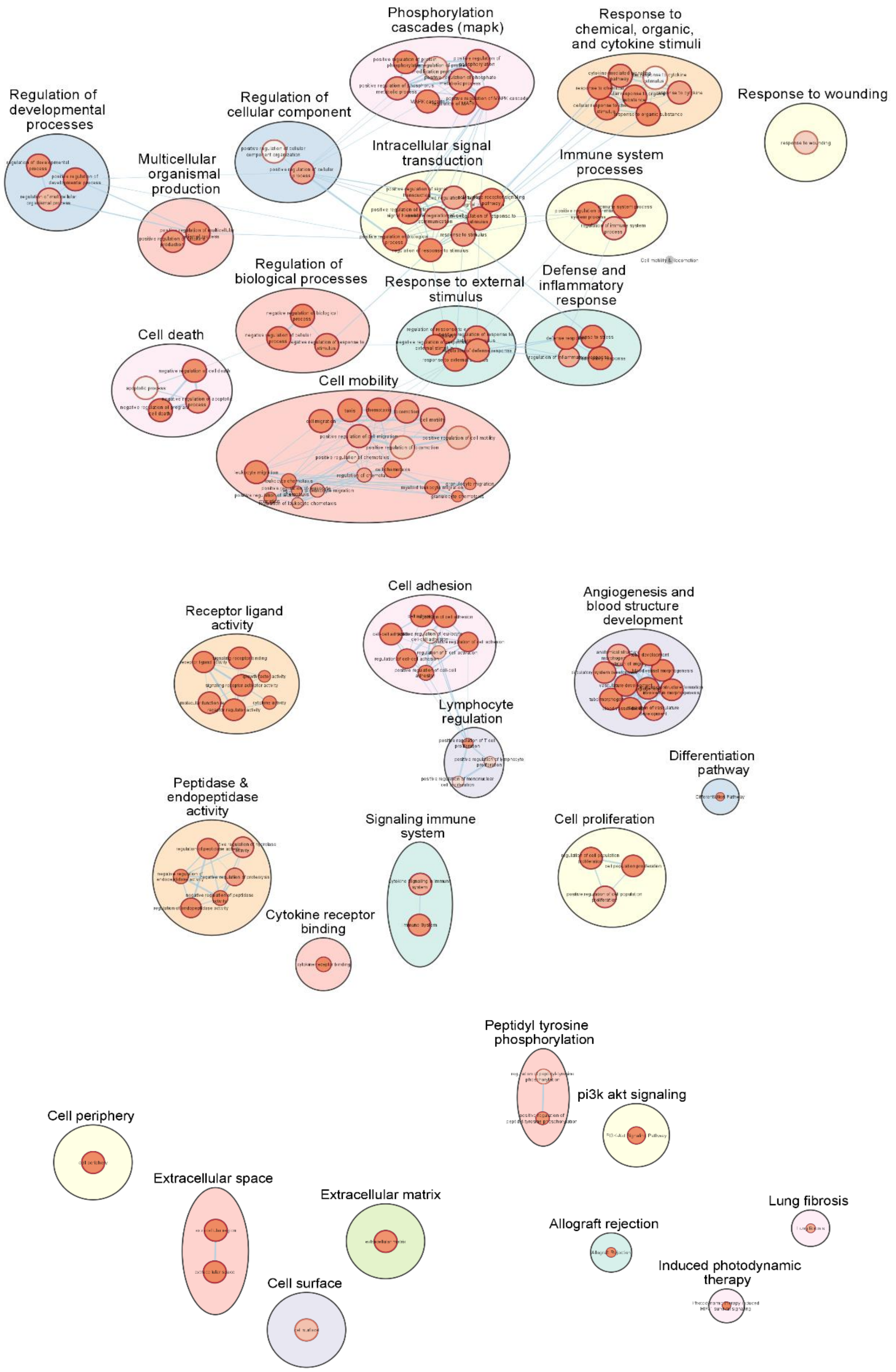

Supplementary Fig. 12: Network of the enriched pathways with the 36 identified markers.

Network analysis with Cytoscape software (using the EnrichmentMap and AutoAnnotate applications). For each group of pathways we list the proteins that are identified in Supplementary Table 2. Source data are provided as a Source Data file.

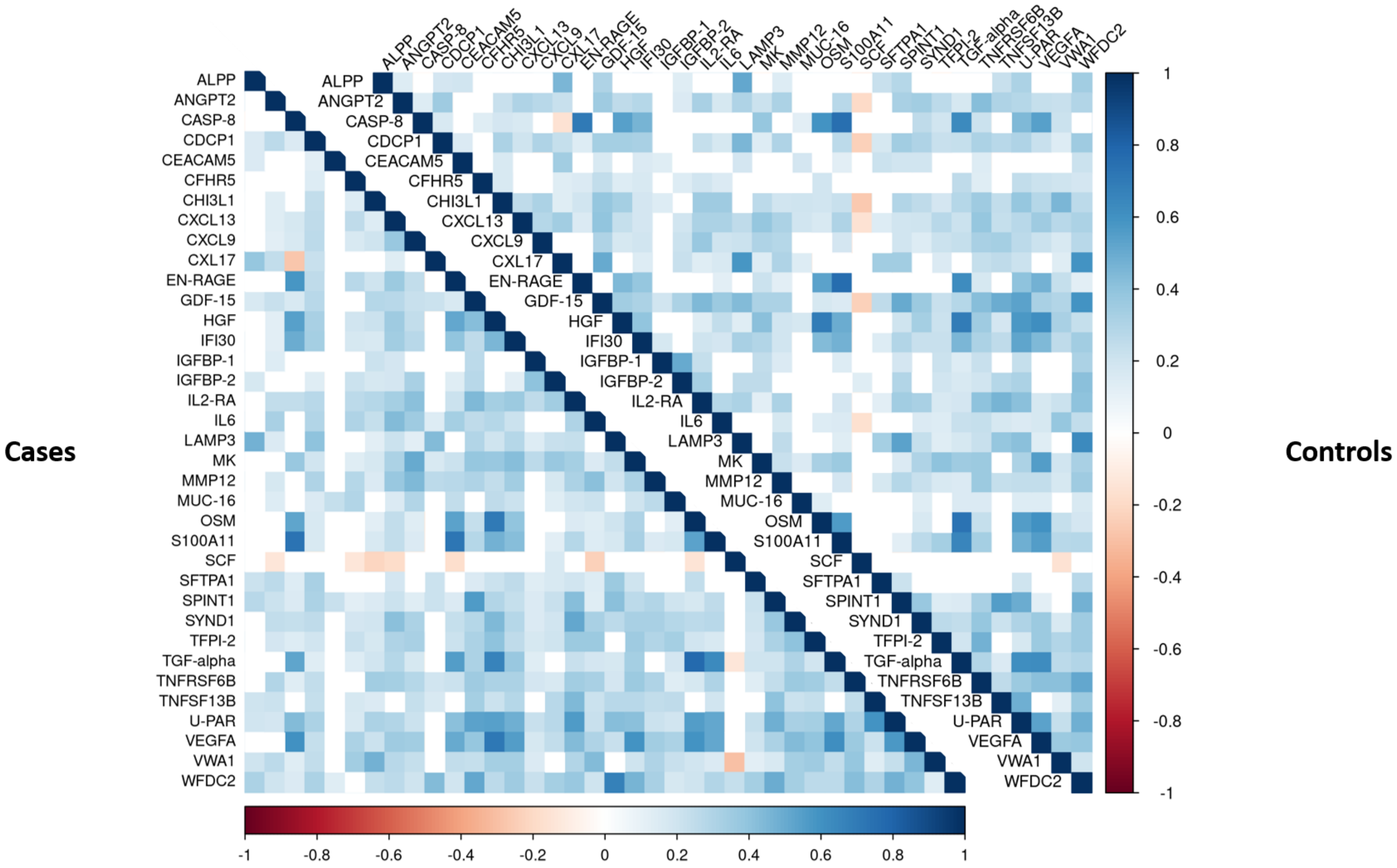

Supplementary Fig. 13: Correlation analyses among 36 proteins associated with imminent lung cancer identified among 731 cases and 731 matched controls in the Lung Cancer Cohort Consortium.

The figure depicts Pearson's correlation coefficients between markers separately in cases (left side) and controls (right side). Pearson's correlation coefficients between markers were estimated after accounting for variation due to sex, age, and cohort (see Supplementary Methods). Source data are provided as a Source Data file.

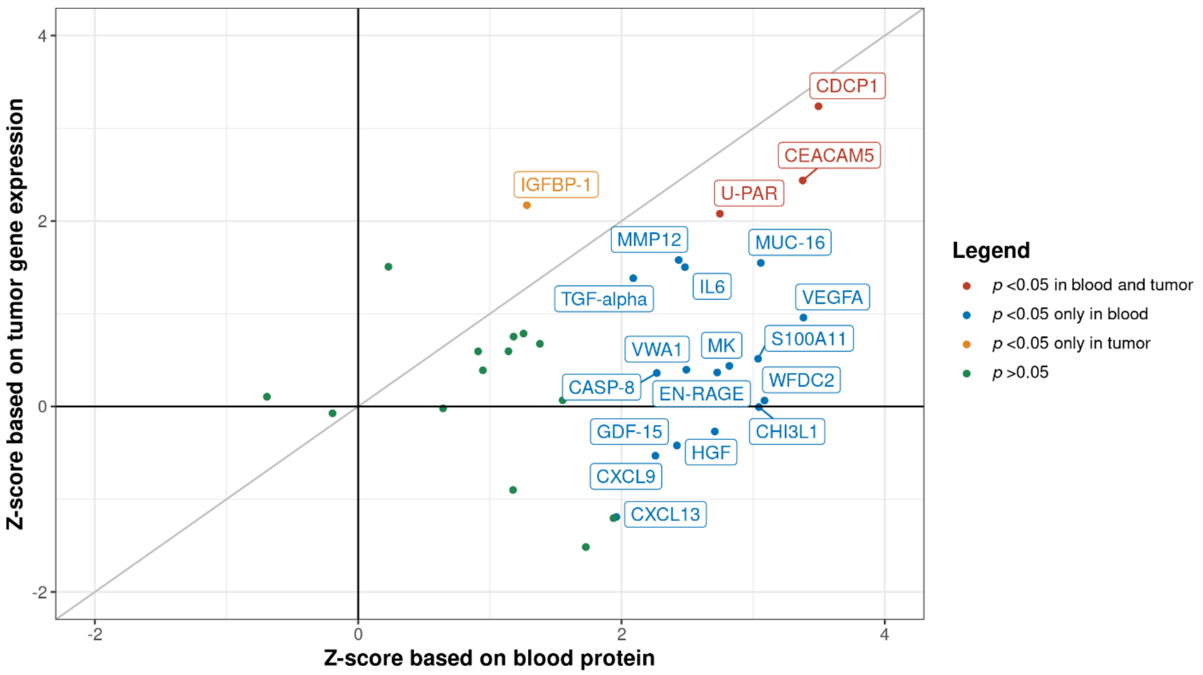

Supplementary Fig. 14: Association between the 36 markers of imminent lung cancer diagnosis and overall mortality among lung cancer cases.

Estimates are based on direct measurements among participants in the Lung Cancer Cohort Consortium (z-scores on x-axis) and tumor gene expression in TCGA (z-scores on y-axis). The grey diagonal line on the figure represents identical Z-scores of the association of the biomarkers with all-cause mortality among lung cancer cases when measured in the blood (x-axis) and when their gene expression is measured in the tumour (y-axis). Source data are provided as a Source Data file.

References

1. Olink Proteomics. https://www.olink.com/.
2. Assarsson, E. *et al.* Homogenous 96-plex PEA immunoassay exhibiting high sensitivity, specificity, and excellent scalability. *PLoS One* **9**, (2014).
3. Robbins, H. A. *et al.* Design and methodological considerations for biomarker discovery and validation in the Integrative Analysis of Lung Cancer Etiology and Risk (INTEGRAL) Program. *Ann. Epidemiol.* (2022) doi:10.1016/j.annepidem.2022.10.014.
4. Patel, H. *et al.* Proteomic blood profiling in mild, severe and critical COVID-19 patients. *Sci. Reports 2021 111* **11**, 1–12 (2021).
5. Bodinier, B., Filippi, S., Nost, T. H., Chiquet, J. & Chadeau-Hyam, M. Automated calibration for stability selection in penalised regression and graphical models: a multi-OMICs network application exploring the molecular response to tobacco smoking. *arXiv:2106.02521* (2021).
6. Csardi, G. & Nepusz, T. The igraph software package for complex network research. *InterJournal* **Complex Sy**, 1695 (2006).
7. Uhlén, M. *et al.* Proteomics. Tissue-based map of the human proteome. *Science (80-.).* **347**, (2015).
8. Uhlen, M. *et al.* A pathology atlas of the human cancer transcriptome. *Science (80-.).* **357**, (2017).
