# Supplementary material for: The blood proteome of imminent lung cancer diagnosis
Source: Nat Commun. 2023 Jun 1;14:3042. doi: 10.1038/s41467-023-37979-8 (PMC10235023; doi:10.1038/s41467-023-37979-8)
Supplement: Supplementary file 2 — Reporting Summary [file 41467_2023_37979_MOESM2_ESM.pdf]

nature portfolio

Corresponding author(s): Hilary A Robbins

Last updated by author(s): Mar 20, 2023

Reporting Summary

Nature Portfolio wishes to improve the reproducibility of the work that we publish. This form provides structure for consistency and transparency in reporting. For further information on Nature Portfolio policies, see our Editorial Policies and the Editorial Policy Checklist.

Statistics

For all statistical analyses, confirm that the following items are present in the figure legend, table legend, main text, or Methods section.

n/a	Confirmed	
☐	☒	The exact sample size (n) for each experimental group/condition, given as a discrete number and unit of measurement
☐	☒	A statement on whether measurements were taken from distinct samples or whether the same sample was measured repeatedly
☐	☒	The statistical test(s) used AND whether they are one- or two-sided<br>*Only common tests should be described solely by name; describe more complex techniques in the Methods section.*
☐	☒	A description of all covariates tested
☐	☒	A description of any assumptions or corrections, such as tests of normality and adjustment for multiple comparisons
☐	☒	A full description of the statistical parameters including central tendency (e.g. means) or other basic estimates (e.g. regression coefficient) AND variation (e.g. standard deviation) or associated estimates of uncertainty (e.g. confidence intervals)
☐	☒	For null hypothesis testing, the test statistic (e.g. F, t, r) with confidence intervals, effect sizes, degrees of freedom and P value noted<br>*Give P values as exact values whenever suitable.*
☒	☐	For Bayesian analysis, information on the choice of priors and Markov chain Monte Carlo settings
☒	☐	For hierarchical and complex designs, identification of the appropriate level for tests and full reporting of outcomes
☐	☒	Estimates of effect sizes (e.g. Cohen's d, Pearson's r), indicating how they were calculated

Our web collection on statistics for biologists contains articles on many of the points above.

Software and code

Policy information about availability of computer code

Data collection	Olink NPX Signature software
Data analysis	All statistical analyses were performed using R version 4.1.2. Packages used: tidyverse, igraph, pROC, survival. Pathway enrichment analysis were done using g:profiler and the Cytoscape software (applications used in Cytoscape were EnrichmentMap and AutoAnnotate,)

For manuscripts utilizing custom algorithms or software that are central to the research but not yet described in published literature, software must be made available to editors and reviewers. We strongly encourage code deposition in a community repository (e.g. GitHub). See the Nature Portfolio guidelines for submitting code & software for further information.

Data

Policy information about availability of data

All manuscripts must include a data availability statement. This statement should provide the following information, where applicable:

- Accession codes, unique identifiers, or web links for publicly available datasets
- A description of any restrictions on data availability
- For clinical datasets or third party data, please ensure that the statement adheres to our policy

The mission of the Lung Cancer Cohort Consortium (LC3) is to facilitate and carry out collaborative research on lung cancer risk and aetiology. The LC3 is committed to facilitating the use of LC3 data by the wider research community for research within its scientific mandate, including: 1- Research on the aetiology of lung cancer incidence and survival. 2- Research on lung cancer risk assessment, early detection, and screening. 3-Research on tobacco exposure and tobacco-related health outcomes. Further details

Access to data from the Lung Cancer Cohort Consortium (LC3) is governed by the LC3 Access Policy, which is available at the following link: https://www.iarc.who.int/wp-content/uploads/2021/12/LC3_Access_Policy.pdf.
Interested investigators are encouraged to contact Dr Johansson or Dr Robbins. We also used TCGA data for gene expression of the identified proteins in lung cancers and publicly available gene expression from the Human Protein Atlas and the Pathology Atlas.

Human research participants

Policy information about studies involving human research participants and Sex and Gender in Research.

Reporting on sex and gender	Some analysis were stratified by sex (biological attributes), as differences in protein levels, and the behavior of lung cancer (outcomes) might be affected it by sex. Sex information was self-reported
Population characteristics	Recruited participants were individuals with a history of smoking (current or former smokers) , with a mean age of 65 years. 33% of the participants were females.
Recruitment	Participants with blood samples at enrollment were identified for this study from previous cohorts. Cases were selected based on the time between their blood draw and diagnosis, and controls were identified to match cases based on age, sex and smoking characteristics
Ethics oversight	IARC Ethics Committee

Note that full information on the approval of the study protocol must also be provided in the manuscript.

Field-specific reporting

Please select the one below that is the best fit for your research. If you are not sure, read the appropriate sections before making your selection.

☒ Life sciences ☐ Behavioural & social sciences ☐ Ecological, evolutionary & environmental sciences

For a reference copy of the document with all sections, see nature.com/documents/nr-reporting-summary-flat.pdf

Life sciences study design

All studies must disclose on these points even when the disclosure is negative.

Sample size	From the full LC3 datasets, we selected 1462 individuals to sample blood from (731 cases and 731 controls). The sample size provides 80% power to identify markers with an ORsd of at least 1.26 after considering multiple testing on all markers analyzed.
Data exclusions	No data was excluded from the analysis.
Replication	Our analysis is based on the replication of results within random splits of the data. We first identified proteins with robust and stable associations across 500 splits of the data (in each iteration the data was split into 70% discovery data where we selected proteins with significant association to lung cancer risk after correction for multiple comparisons , and 30% replication where the selected proteins had to have at least nominal -p<0.05- significance in order to be labeled as a marker of prediagnostic lung cancer). The ORs presented are calculated in the full dataset.
Randomization	We use a matched case-control design, therefore randomization is not applicable.
Blinding	We use anonymized data

Reporting for specific materials, systems and methods

We require information from authors about some types of materials, experimental systems and methods used in many studies. Here, indicate whether each material, system or method listed is relevant to your study. If you are not sure if a list item applies to your research, read the appropriate section before selecting a response.

Materials & experimental systems

n/a	Involved in the study
☒	☐ Antibodies
☒	☐ Eukaryotic cell lines
☒	☐ Palaeontology and archaeology
☒	☐ Animals and other organisms
☒	☐ Clinical data
☒	☐ Dual use research of concern

Methods

n/a	Involved in the study
☒	☐ ChIP-seq
☒	☐ Flow cytometry
☒	☐ MRI-based neuroimaging
