# Supplementary material for: The blood proteome of imminent lung cancer diagnosis
Source: Nat Commun. 2023 Jun 1;14:3042. doi: 10.1038/s41467-023-37979-8 (PMC10235023; doi:10.1038/s41467-023-37979-8)
Supplement: Supplementary file 3 — Description of Additional Supplementary Files [file 41467_2023_37979_MOESM3_ESM.docx]

**Description of Additional Supplementary Files**

**Supplementary Data 1:** Characteristics of 731 lung cancer cases and 731 matched controls stratified by cohort.

**Supplementary Data 2a and Supplementary Data 2b:** Quality controls of assay measures in the EPIC and NSHDS cohorts (1a), and in in the CPS, HUNT, MCCS and SCHS cohorts (1b).

Footnote: Follow-up time for lung cancer may be shorter than follow-up time for mortality due to different end dates for the completeness of cancer registry vs mortality registry data.

**Supplementary Data 3:** Proportion of samples below the LOD.

**Supplementary Data 4:** Observed effect size of all measured proteins with lung cancer risk in the full data.

**Supplementary Data 5:** Proportion of 500 random discovery-replication samples in which risk-associated proteins were replicated.

**Supplementary Data 6:** Comparison of the estimated associations between each protein and lung cancer risk identified by the single split design vs the resampling algorithm.

**Supplementary Data 7:** Stratified associations of the 36 identified markers with lung cancer risk & AUCs across stage strata.

**Supplementary Data 8:** Stratified associations of the 36 identified markers with lung cancer risk across different strata.

**Supplementary Data 9:** Trends by lead time in the association between the 36 identified markers and lung cancer risk.

**Supplementary Data 10:** Lung cancer odds ratios for the 36 proteins associated with imminent lung cancer before and after detailed adjustment for smoking intensity and duration.

Footnote: we defined lead time as the time (in years) elapsed between blood draw and clinical diagnosis of lung cancer.

**Supplementary Data 11:** Centralities of the penalized networks of the 36 identified markers.

Footnote: Degree centrality represents the number of edges each node has (i.e. the number of proteins each protein is directly connected to).

Betweenness centrality represents the importance of each node to the flow of the network by assessing the number of short paths between two nodes each node is on (i.e. if protein A is connecting protein B and C and there’s no other link to B and C, then A would have a high betweenness centrality. If protein D is connected to B and has no other connection, D is therefore not linking any proteins and will have a low betweenness centrality).

Closeness centrality represents the average distance between each node and the other nodes (i.e. for each protein we calculate the inverse of the sum of the distances to every other protein). The higher the closeness is, the more each protein is efficiently related to the other proteins in the network.

Eigen vector centrality is an extension of degree centrality. It adjusts the centrality degree assigned by the number of direct links to each protein for their “power”. In other words, if protein A and protein B are each independently linked to 5 other proteins then both have a high degree centrality. However if the 5 proteins linked to protein A have no importance in the network (are not linked to other proteins) while the 5 proteins linked to B are linked to other proteins in the network than protein B will have a higher eigenvector centrality than protein A.

**Supplementary Data 12:** Association between the 36 markers of imminent lung cancer diagnosis and overall mortality among lung cancer cases, based on direct measurements among participants in the Lung Cancer Cohort Consortium and tumor gene expression in TCGA.

**Supplementary Data 13:** Vital status outcomes among lung cancer cases.
